# Functional analysis of *Plasmodium falciparum* subpopulations associated with artemisinin resistance in Cambodia

**DOI:** 10.1186/s12936-017-2140-1

**Published:** 2017-12-19

**Authors:** Ankit Dwivedi, Christelle Reynes, Axel Kuehn, Daniel B. Roche, Nimol Khim, Maxim Hebrard, Sylvain Milanesi, Eric Rivals, Roger Frutos, Didier Menard, Choukri Ben Mamoun, Jacques Colinge, Emmanuel Cornillot

**Affiliations:** 1 Institut de Biologie Computationnelle (IBC), 34095 Montpellier, France; 2 Institut de Recherche en Cancérologie de Montpellier, Institut régional du Cancer Montpellier & Université de Montpellier, IRCM-INSERM U1194, 34298 Montpellier, France; 3 Laboratoire de Biostatistiques, Informatique et Physique Pharmaceutique, UFR Pharmacie, Université de Montpellier, 34093 Montpellier, France; 4 Institut de Génomique Fonctionnelle-CNRS, 34094 Montpellier, France; 5 Centre de Recherche en Biologie cellulaire de Montpellier, CNRS-UMR 5237, 34293 Montpellier, France; 6 Malaria Molecular Epidemiology Unit, Institut Pasteur du Cambodge, Phnom Penh, Cambodia; 7 Laboratoire d’informatique, de robotique et de microélectronique de Montpellier, LIRMM, CNRS, Université de Montpellier, 34095 Montpellier, France; 8 Present Address: Center for Integrative Medical Sciences, RIKEN, Yokohama, Kanagawa Japan; 9 CIRAD, UMR Intertryp, 34398 Montpellier, France; 10 IES, UMR 5214, Université de Montpellier, CNRS, 34095 Montpellier, France; 11 Present Address: Biology of Host-Parasite Interactions Unit, Institut Pasteur, Paris, France; 12 Section of Infectious Diseases, Department of Internal Medicine, Yale School of Medicine, New Haven, CT 06520 USA; 13 Present Address: Institute for Genome Sciences, University of Maryland School of Medicine, Baltimore, MD 21201 USA

**Keywords:** *Plasmodium falciparum*, Malaria, Cambodia, Artemisinin resistance, *k13*, Population fragmentation, Admixed subpopulations, Network based stratification, Shifting balance theory, Redox metabolism

## Abstract

**Background:**

*Plasmodium falciparum* malaria is one of the most widespread parasitic infections in humans and remains a leading global health concern. Malaria elimination efforts are threatened by the emergence and spread of resistance to artemisinin-based combination therapy, the first-line treatment of malaria. Promising molecular markers and pathways associated with artemisinin drug resistance have been identified, but the underlying molecular mechanisms of resistance remains unknown. The genomic data from early period of emergence of artemisinin resistance (2008–2011) was evaluated, with aim to define *k13* associated genetic background in Cambodia, the country identified as epicentre of anti-malarial drug resistance, through characterization of 167 parasite isolates using a panel of 21,257 SNPs.

**Results:**

Eight subpopulations were identified suggesting a process of acquisition of artemisinin resistance consistent with an emergence-selection-diffusion model, supported by the shifting balance theory. Identification of population specific mutations facilitated the characterization of a core set of 57 background genes associated with artemisinin resistance and associated pathways. The analysis indicates that the background of artemisinin resistance was not acquired after drug pressure, rather is the result of fixation followed by selection on the daughter subpopulations derived from the ancestral population.

**Conclusions:**

Functional analysis of artemisinin resistance subpopulations illustrates the strong interplay between ubiquitination and cell division or differentiation in artemisinin resistant parasites. The relationship of these pathways with the *P. falciparum* resistant subpopulation and presence of drug resistance markers in addition to *k13*, highlights the major role of admixed parasite population in the diffusion of artemisinin resistant background. The diffusion of resistant genes in the Cambodian admixed population after selection resulted from mating of gametocytes of sensitive and resistant parasite populations.

**Electronic supplementary material:**

The online version of this article (10.1186/s12936-017-2140-1) contains supplementary material, which is available to authorized users.

## Background

Malaria is one of the widespread parasitic infections affecting humans and World Health Organization malaria elimination programmes are threatened by the emergence of drug resistance. Cambodia is considered to be the epicentre of *Plasmodium falciparum* resistance to the first-line treatment, artemisinin-based combination therapy [1]. The emergence and existence of artemisinin resistant parasites is now reported at different locations in Greater Mekong Subregion (GMS) [2–10]. Understanding the mechanism underlying resistance is crucial for the identification of new drug targets and restriction of spread of artemisinin resistance to high malaria transmission areas such as Africa.

The fragmentation of Cambodian *P. falciparum* population into artemisinin resistant (founder subpopulations), sensitive (ancestral population) and admixed subpopulations was shown in recent reports [11–13]. Furthermore, four non-synonymous mutations (C580Y, Y493H, R539T and I543T) in the propeller domain of the Kelch gene *k13* (*PF3D7_1343700*), located on chromosome 13, were described as determinants of clinical artemisinin resistance in Cambodia [14]. Nine other non-synonymous mutations in the *k13* were associated with clinical artemisinin resistance in GMS [2–10]. The *k13* mutant alleles exhibiting clinical artemisinin resistance have not been observed outside the GMS [10]. Some of the *k13* artemisinin resistant alleles have emerged independently with different geographical origins in different locations in SEA [4, 8] and have spread to neighboring countries [15]. This could be due to different selective pressure at different locations [10]. The most dominant and transmissible allele reaching fixation is observed to be C580Y in Cambodia, Thailand–Myanmar border, Vietnam and southern Laos, with a single origin in western Cambodia [8–10, 15, 16]. There could be certain essential genetic background mutations facilitating the artemisinin drug resistance through selection, a similar phenomenon observed with the resistance to chloroquine, pyrimethamine or sulfadoxine in the 1960s in the GMS [1, 17]. Emergence was also suspected to be related to the small and well-structured parasite population in the GMS [18] The relative low transmission preventing the development of protective immunity in the human population may have helped emergence of drug resistance, especially at the border between Cambodia and Thailand [19]. The other reason for emergence of resistance could be the early introduction of anti-malaria drug based treatments, with inappropriate dose or usage that may have led to continual drug exposure of malarial parasite populations [20, 21].

Based on genome wide analysis, four genes *Pffd*, *Pfcrt*, *Pfarps10* and *Pfmdr2*, have been described as background genes supporting *k13* resistant alleles [4]. Although, these markers certainly determine artemisinin resistance, the underlying resistance mechanism is still unknown. Mbengue and colleagues have described a K13-PI3K pathway to be potentially associated with artemisinin resistance, and that PI3K could be targeted for understanding resistance mechanism [22]. Also, biological process and pathways like ubiquitination, oxidative stress response and unfolded protein response pathways, are associated with artemisinin resistance [23, 24]. The present study aims to associate the biological processes and metabolic pathways with the early *P. falciparum* population structure in Cambodia, by analysing mutations in parasites isolated in Cambodia during the early period of emergence of artemisinin resistance (2008–2011). An integration of population structure, genetic variations, networks and annotation data was performed using a theory described here as emergence-selection-diffusion (ESD) model. Bioinformatic results on significant mutations and related biological pathways were used to test the model and evaluate the process of emergence and selection in specific small subpopulations, followed by diffusion in an admixed population. This model defined for a descriptive approach is similar to the population shifting balance and metapopulation theories in population genetics that were successfully implemented for the analysis of *P. falciparum* populations [25, 26].

In total, 21,257 non-synonymous SNPs were identified using specific filters among 167 isolates from the genomic dataset on which the parasite population structure was described earlier [12]. Based on hierarchical clustering and network-based stratification [27], eight specific subpopulations were identified among four regions in Cambodia. An extended list of 57 background genes associated with artemisinin resistant parasites in Cambodia is described. In addition to the identification of known targets, functional analysis reveals a strong interplay between ubiquitination and cell division in artemisinin resistant parasites.

## Methods

### Data acquisition

The genome sequencing data of Cambodian parasite isolates was recovered from the ENA (European Nucleotide Archive) database server, submitted by the Welcome Trust Sanger Institute. Out of the 293 *P. falciparum* whole genome sequences from 2008 to 2011, which were used to define the *P. falciparum* parasite population structure in Cambodia [11, 12], 167 sequences were recovered successfully (Additional file 1) in BAM format and converted to much readable VCF (v4.1) files [28] using SAMtools v0.1.19 [29]. *Plasmodium falciparum* 3D7 strain (genome sequence annotation version 2) was used as the reference genome, and was recovered from the PlasmoDB Plasmodium Genomics resource database (release version 5.5) [30]. The 167 genome sequences originate from four locations: Pailin (14 sequences), Tasanh (26 sequences) and Pursat (81 sequences) in western Cambodia and Ratanakiri (46 sequences) in eastern Cambodia.

### Filtering data for noise

A reliable variant calling pipeline was established for the identification of significant SNPs (Additional file 2). The analysis was focused on non-synonymous SNPs in the coding region of the genome, which occur in at least one of the isolates, and does not take insertion/deletion (INDELs) into consideration. The SNP data was filtered for noise based on the VCF (v4.1) file signal parameters. For identification of thresholds on these parameters to filter the data, at first, around 100 kb were removed from starting and end of each chromosome. These 100 kb regions at the extremity of *P. falciparum* chromosomes encode for multi-gene families, most of them encoding putative surface antigens (including VAR, Rifin, Stevor). An average value for all the signal parameters over 167 isolates were calculated for each SNP position as sum of the values in different isolates over the number of isolates having a non-reference allele. The average quality values per position were plotted along the genome, and a clear threshold in the mapping quality (MQ) parameter was observed at value 29 (Additional file 3). Removing chromosome ends removed most of the signal below the score 29. Therefore, mapping quality score higher than 29 was considered as one of the filtering criteria. Many SNPs showed MQ ≤ 29 in the coding core of chromosome 4 and 7, as internal VAR gene clusters are present in these chromosomes. No other quality parameters plotted along the genome, with or without the chromosome end regions showed any change in distribution of SNPs, on which a significant threshold could be defined. In order to select the SNPs with high quality non-reference (non-REF) signal, DP4, the parameter accounting for high-quality forward strand REF bases, reverse strand REF bases, forward strand non-REF bases and reverse strand non-REF bases for a specific position was analysed. To account for the percentage of non-REF signal (referred to as DA in the manuscript) and choose a threshold, the distribution of proportion of non-REF alleles (forward strand non-REF bases + reverse strand non-REF bases) over the sum of all alleles was analysed. The value 0.7 was chosen, as it seemed to be the intersection of two distributions: low quality SNPs on the left and high quality SNPs on the right (Additional file 4). Therefore, DA score ≥ 0.7 was considered as the second filtering criteria to include SNPs with high quality non-REF allele calls. Around 20,000 SNPs were recovered for each isolate after implementing these two filters (min: 13,470, max: 23,022 SNPs). There were 247,783 SNPs having a non-REF allele in at least one of the 167 Cambodian parasite genomes.

In order to remove the SNPs with rare allele frequencies in the population, minor allele frequency (MAF) for each SNP was analysed (Additional file 5). It is defined as the minimum of non-reference allele frequency (NRAF) and 1-NRAF [11, 31]. All the SNPs with a minor allele frequency (MAF) less than 0.01796 were removed from the analysis (Additional file 5). After applying the three filters, 111,701 unique SNPs were recovered in 167 Cambodian isolates.

### Correspondence between different genome versions

The correspondence of the SNP coordinates between *P. falciparum* genome sequence version 2 and 3 was recovered using BLAST (Basic Local Alignment Search Tool) from NCBI (National Centre for Biotechnology Information), for each chromosome separately. For the chromosomes 4, 7, 8, 10 and 13 very short lengths of alignments were obtained depicting major changes in the genomic sequence between version 2 and 3. Correspondence for these chromosomes was then obtained by defining specific regions for BLAST. Correspondence for approximately all SNPs in all chromosomes was recovered, and the 3105 unmapped SNPs in the recovered dataset were removed from the analysis. NCBI BLAST was performed for each chromosome, with genome version 2 (PlasmoDB release version 5.5) as reference and version 3 (PlasmoDB release version 10) as query, to recover a list of 108,596 SNPs.

### Removing uncertain SNPs and correcting errors

After filtering the data and removing the unmapped SNPs, the uncertainties were treated. SNPs with more than one ALT (non-REF) allele for a specific isolate were considered as uncertainties. SNPs having the uncertain ALT allele frequency higher than 40% were removed (830 SNPs). For the cases where SNPs had only one ALT allele in most of the isolates and uncertain ALT allele in some isolates, the uncertain allele was substituted with the ALT value (16,859 SNPs). For the other cases where SNPs had more than one ALT allele for different isolates, the uncertain ALT alleles were substituted with the most frequent ALT allele (1772 SNPs). The ALT alleles with the frequency 1.5 times the frequency of same allele at random, are considered as the most frequent ALT allele for SNP. In the case of uncertain ALT allele frequency less than 5% and no majority ALT allele, the uncertain ALT allele was substituted with the REF allele (54 SNPs). All the other cases were removed from the analyses (1228 SNPs). A total of 106,538 unique SNPs were recovered in 167 Cambodian isolates with 18,683 modified SNPs (Additional file 6).

### Annotation of the recovered SNPs

To describe the distribution of the SNPs over the genome, the 167 strains were annotated using VCF-annotator Perl script (developed at the Broad Institute, Cambridge, MA). The GFF3 (General Feature Format version 3) file was recovered from Ensembl database server (release version ASM276v1.21) corresponding to the *P. falciparum* genome sequence version 2. The annotation shows that the recovered SNPs are mostly in introns, exons and 5pUTR. To define the coordinates of the chromosome end regions the chromosomes corresponding to genome version 3 (PlasmoDB release version 11) were visualized using the genome browser provided by PlasmoDB. The regions containing the genes with descriptions such as CLAG, DBL, Rifin, hyp, Stevor, GARP, RESA, VAR, PfEMP, Surfin, PHIST, KAH and EMP in a consecutive organization, were considered as chromosome end regions. The gene location in the coding region was determined and chromosome end coordinates were defined (Table 1).

**Table 1:** Chromosome end coordinates

Chromosome	Positions excluded from start of chromosome	Positions excluded from end of chromosome	Chromosome length (bp)
Chromosome 1	1–117,000	481,500–640,851	640,851
Chromosome 2	1–120,000	783,000–947,102	947,102
Chromosome 3	1–135,000	1,002,000–1,067,971	1,067,971
Chromosome 4	1–174,000	1,067,000–1,200,490	1,200,490
Chromosome 5	1–49,000	1,297,000–1,343,557	1,343,557
Chromosome 6	1–74,000	1,293,000–1,418,242	1,418,242
Chromosome 7	1–91,000	1,320,000–1,445,207	1,445,207
Chromosome 8	1–90,000	1,296,000–1,472,805	1,472,805
Chromosome 9	1–127,000	1,380,000–1,541,735	1,541,735
Chromosome 10	1–112,000	1,515,000–1,687,656	1,687,656
Chromosome 11	1–138,000	1,934,000–2,038,340	2,038,340
Chromosome 12	1–98,000	2,130,000–2,271,494	2,271,494
Chromosome 13	1–129,000	2,808,000–2,925,236	2,925,236
Chromosome 14	1–71,000	3,129,000–3,291,936	3,291,936

This table represents the coordinates at the start (column 2) and the coordinates at the end (column 3) for each chromosome (column 1) that are not included in the analysis. The size of different chromosomes as in *P. falciparum* 3D7 genome version 3 (PlasmoDB release version 11) is mentioned in column 4

The final set of 21,257 non-synonymous SNPs in the coding region was described, using the recovered annotation and chromosome ends region coordinates (Additional file 7). This dataset is referred to as IBC dataset in the manuscript and is used for parasite population study in Cambodia. There are 3714 modified SNPs (as described in the section above) in the set of these 21,257 SNPs.

### Clustering

To describe the *P. falciparum* population structure in Cambodia, unsupervised hierarchical clustering was performed on the IBC dataset (all the statistical analysis is performed in R v3.0.1 and v3.2.3). The pairwise distance between two isolates was estimated as the proportion of base substitution between them over the whole set of recovered SNPs. Ward minimum variance method was used as a metric to build the dendrogram. The correspondence between previously described parasite subpopulations in Cambodia [12] and the 167 isolates were recovered from the Sanger Institute. Eight subpopulations were described based on the hierarchal clustering results: KH1.1, KH1.2, KH2.1, KH2.2, KH3, KH4, KH5 and KHA. In order to choose the optimal number of clusters in the dendrogram, the value of k was set to 2 to 10 and the clusters obtained at k = 8 overlapped both, clusters based on different *k13* alleles and the previously described KH subpopulations. By further increasing the k, only the admixed subpopulation KHA was further divided into small subpopulations.

### Significant SNPs and genes

To describe the metabolic pathways and functions associated with different subpopulations, significant genes were recovered based on significant SNPs. For each subpopulation significant SNPs were defined using one-tailed Fisher-exact test, by comparing the ALT allele frequency of each SNP in each subpopulation to the to the ALT allele frequency in artemisinin sensitive population KH1.1, which is considered as the ancestral population [12]. Only the SNPs with ALT allele increased frequency in a particular subpopulation were considered. The Benjamini–Hochberg method was used to correct the *p* values from multiple comparisons. All the SNPs with corrected *p* value lower than 0.05 were considered as significant and the genes containing these significant SNPs were defined as the significant genes (Table 2).

**Table 2:** Number of significant SNPs and genes in each subpopulation compared to ancestral artemisinin sensitive subpopulation

Subpopulations	Number of isolates	Number of significant SNPs	Number of significant genes
KH1.2	5	1361	823
KH2.1	11	1620	938
KH2.2	22	2312	1125
KH3	12	1495	859
KH4	9	1891	978
KH5	14	1612	900
KHA	49	740	493

Significant SNPs were described using one tailed Fisher-exact test. For each SNP the ALT frequency in each subpopulation was compared to the ALT frequency in KH1.1 (45 isolates) to calculate the *p* value. SNPs with *p* value ≤ 0.05 were considered as significant and the genes containing these SNPs are considered as the significant genes in each subpopulation

### Gene interaction networks and gene ontology

In order to determine the biological processes associated with different subpopulations, gene–gene interaction networks were analysed. The interaction network data was recovered from STRING v10, which provides functional and predicted protein–protein interactions from other publicly available data sources and literature [32]. Networks based on co-expression data for all the subpopulations based on significant genes were recovered. Only edges with a STRING confidence score for co-expression higher than 0.5 were considered for the analysis. The networks were imported and analysed in Cytoscape v3.2.1 [33].

The complete *P. falciparum* interaction network based on co-expression data (confidence score ≥ 0.5) was also recovered from STRING v10 database. Out of the 5777 genes identified in the genome of *P. falciparum* (PlasmoDB release v24), only 3875 genes had co-expression interaction confidence score greater than 0.5. Out of these 3875 genes, 33 genes did not have interactions with the major interaction network and are not considered in this analysis. The 3842 genes were classified into six parasite intra-erythrocytic stage forms (early ring, late ring, early trophozoite, late trophozoite, early schizogony and late schizogony). These genes were classified according to the maximum expression stage data, which was based on microarray transcriptomic data of the study by Le Roch et al. [34], available in PlasmoDB server (“Pf-iRBC + Spz + Gam Max Exp Timing” column). The genes with maximum expression in merozoite intra-erythrocyte stage of the parasite were not focused (711 genes). Also, 138 genes were not classified into any blood stage according to the maximum expression stage data. The remaining 2993 genes were majorly distributed into ring and trophozoite, followed by schizont blood stage (Table 3).

**Table 3:** Number of genes in *P. falciparum* gene–gene interaction network with maximum expression in different parasite blood stage forms

Gene classification	Number of genes	
Ring		
Early	702	1043
Late	341	
Trophozoite		
Early	548	1082
Late	534	
Schizogony		
Early	391	868
Late	477	
Merozoite	711	
Unclassified	138	
Total	3842	

This table is based on the co-expression interaction network (confidence score ≥ 0.5) recovered from STRING v10 database server. The unconnected nodes (genes) were not considered in the analysis. The maximum expression data is based on the transcriptomic study by Le Roch et al. [34] and is recovered from PlasmoDB. Data for early and late forms of the three blood stage ring, trophozoite and schizogony is available. Some of the genes are not classified in the interaction network

For identification of biological function associated to the significant genes, the functionally grouped networks of GO terms and pathways were recovered and analysed for each subpopulation using the ClueGO v2.2.4 [35] and CluePedia v1.2.4 [36] plugins of Cytoscape. The network is created with nodes as the term and edges as the association based on kappa score. All the default conditions of the ClueGO plugin were used. Right-sided hypergeometric test (enrichment) was used as the statistical test and the Benjamini–Hochberg method was used to correct *p* values.

### Network based subpopulation description

The population structure of 167 parasite isolates was also questioned using the method of Network Based Stratification [27] which clusters the isolates together having diffusion paths associated to mutated genes in similar network regions. The list of mutated genes for each isolate was projected on the full *P. falciparum* interaction network recovered from STRING v10 [32]. All prediction sources such as co-expression, co-occurrence, gene fusion, databases, experimental evidence, text-mining and neighbourhood were considered for the full *P. falciparum* interaction network. The results were obtained for the top 10% gene–gene interactions based on combined confidence score provided by STRING database [32]. The mutated genes were propagated to the neighbourhood network (network smoothing) and based on the node score matrix of the resulting diffusion network for each isolate, clustering was performed using non-negative matrix factorization (NMF) method [37]. Consensus clustering was performed by selecting 80% mutated genes and 80% isolates 100 times randomly and iterating NMF clustering 10 times. The consensus clustering between samples was estimated as the percentage of co-clustering results in which they are in the same cluster when the dendrogram is cut to obtain 8 groups (to make the comparison easy with the 8 KH sub-populations). This consensus clustering matrix was normalized and then used to build a dendrogram for all 167 isolates using Euclidean distance matrix and ward minimum variance method in R.

Some of the isolates were grouped in different clusters, compared to the clustering results based on 21,257 SNPs. There were 10 isolates (isolate index: 9, 70, 91, 103, 112, 134, 148, 151, 160, 162) clustering in KHA (based on 21,257 SNPs), but according to clustering based on network based stratification these isolates clustered in other resistant and sensitive subpopulations. Also one isolate (isolate index: 156) was classified in KH2.2 which was in KH2.1 earlier and one isolate (isolate index: 61) is classified in KH2.1 which was in KH3 earlier. All the other 155 isolates completely overlaps with the clusters observed previously.

### Ribosome S10 protein structure prediction

Models were built using four independent structure prediction servers, IntFOLD [38], Phyre2 [39], RaptorX [40] and LOMETS [41]. For each model, the top model from each of the four servers was analysed using ModFOLDclust2 [42] and manually inspected, showing all models had the same fold. The RaptorX models were selected as the best representative models according the ModFOLDclust2 score and manual inspection. Structural superposition of the RaptorX models was undertaken using the TM-align method [43], which produces a TM-score between 0 and 1, with scores above 0.5 indicating the same fold and scores close to 1 indicating a high degree of structural similarity of the two proteins.

## Results

### Description of *P. falciparum* subpopulations

**Fig. 1:**
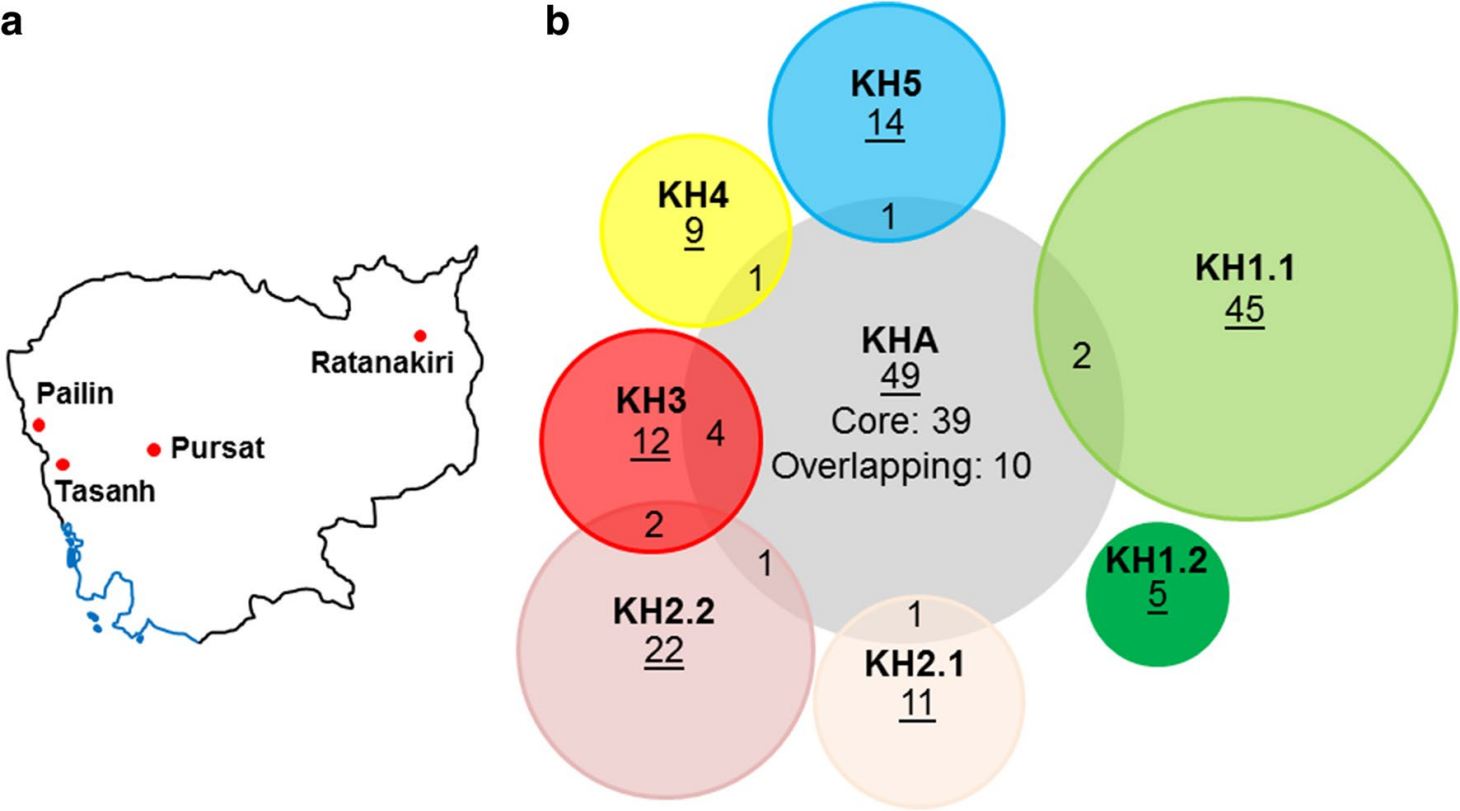
*Plasmodium falciparum* population structure in Cambodia during early period of emergence of artemisinin resistance (2008–2011). **a** Map of Cambodia and localization of health center (red dots) from where the 167 samples originate. **b** Eight groups were identified by hierarchical clustering based on 21,257 non-synonymous SNPs to classify 167 samples. Naming of subpopulations was performed according to previous description of the *P. falciparum* parasite population structure [12], and the overlap with earlier classification was around 90%. KH5 ART-R subpopulation was introduced. The KH1 and KH2 subpopulations [12] were split in two groups. Group structure was confirmed by network based stratification method, based on co-expression data. Ten samples from the admixed subpopulation KHA were differentially classified (overlap between disks). Small overlap was found between KH3 and KH2.2. One sample in each subpopulation was found in the other group. Surface of the disks is proportional to the number of samples (underlined characters). The color code is reminiscent of the major *k13* alleles found in the subpopulation: shades of green: WT, blue: R539T, yellow: Y493H, and shades of red: C580Y

Identification of *P. falciparum* subpopulations from 2008 to 2011 Cambodian samples revealed specific origin of artemisinin resistant *k13* alleles. Hierarchical clustering was performed based on 21,257 non-synonymous SNPs (Additional file 7) to classify 167 samples into eight subpopulations (Fig. 1; Additional file 8). The *P. falciparum* parasite population structure described earlier [12] was confirmed and refined. Isolates carrying one of the three dominant *k13* alleles C580Y, Y493H and R539T found in the dataset are referred to as ART resistant isolates and the five subpopulations of which nearly all the isolates were carrying the *k13* alleles are referred to as artemisinin resistant (ART-R) subpopulations (Additional file 8). The subpopulations KH2 and KH3 are the donor populations for the most dominant allele C580Y (the isolates in KH2 are classified into two subpopulations KH2.1 and KH2.2). One isolate in KH2.2 does not carry any of the *k13* mutant alleles. KH4 is the donor population for the Y493H allele. KH5 is a new subpopulation which was not described earlier, with all the isolates in this subpopulation carrying any one of the three *k13* alleles in a mutually exclusive fashion. R539T is the dominating allele followed by C580Y and Y493H alleles. Hence, KH5 is considered to be a donor population for R539T allele of the *k13* gene. Two KH5 isolates match with a genotype barcode that has been associated with mefloquine resistance [13]. The ART sensitive (ART-S) subpopulation KH1 described as the ancestral population [12] was classified into two subpopulations, KH1.1 and KH1.2. None of the isolates in these two subpopulations carry any of the three *k13* alleles and most of the isolates originate from Ratanakiri in eastern Cambodia (Additional file 8). KH1.1 is one of the largest subpopulations and KH1.2 is the smallest subpopulation. The other large subpopulation is the admixed subpopulation KHA with around 52% of the ART-R isolates and 48% ART-S isolates.

The parasite population structure in Cambodia was also confirmed using a stratification method [27] based on co-expression network data recovered from STRING database [32]. Only some admixed subpopulation KHA samples were differentially classified, suggesting potential close relationship with the donor subpopulations in terms of gene expression (Additional file 9). Consequently, out of the 49 isolates in this subpopulation, 10 isolates were clustering with other subpopulations (Fig. 1b). The clustering of 6 mutant *k13* and 4 wildtype *k13* isolates with the ART-R donor subpopulations was intriguing regarding the diffusion of *k13* alleles after mating between gametocytes. For example, no ART-R isolates were found with a KH1 background of gene expression. Furthermore, clustering shows that the KH1.2 ART-S subpopulation is robust and gene expression network might be closer to ART-R subpopulations than ART-S subpopulation KH1.1, suggesting the presence of *P. falciparum* parasite subpopulations with specific genetic backgrounds in Cambodia before the introduction of ART.

### Subpopulation associated genetic background

**Fig. 2:**
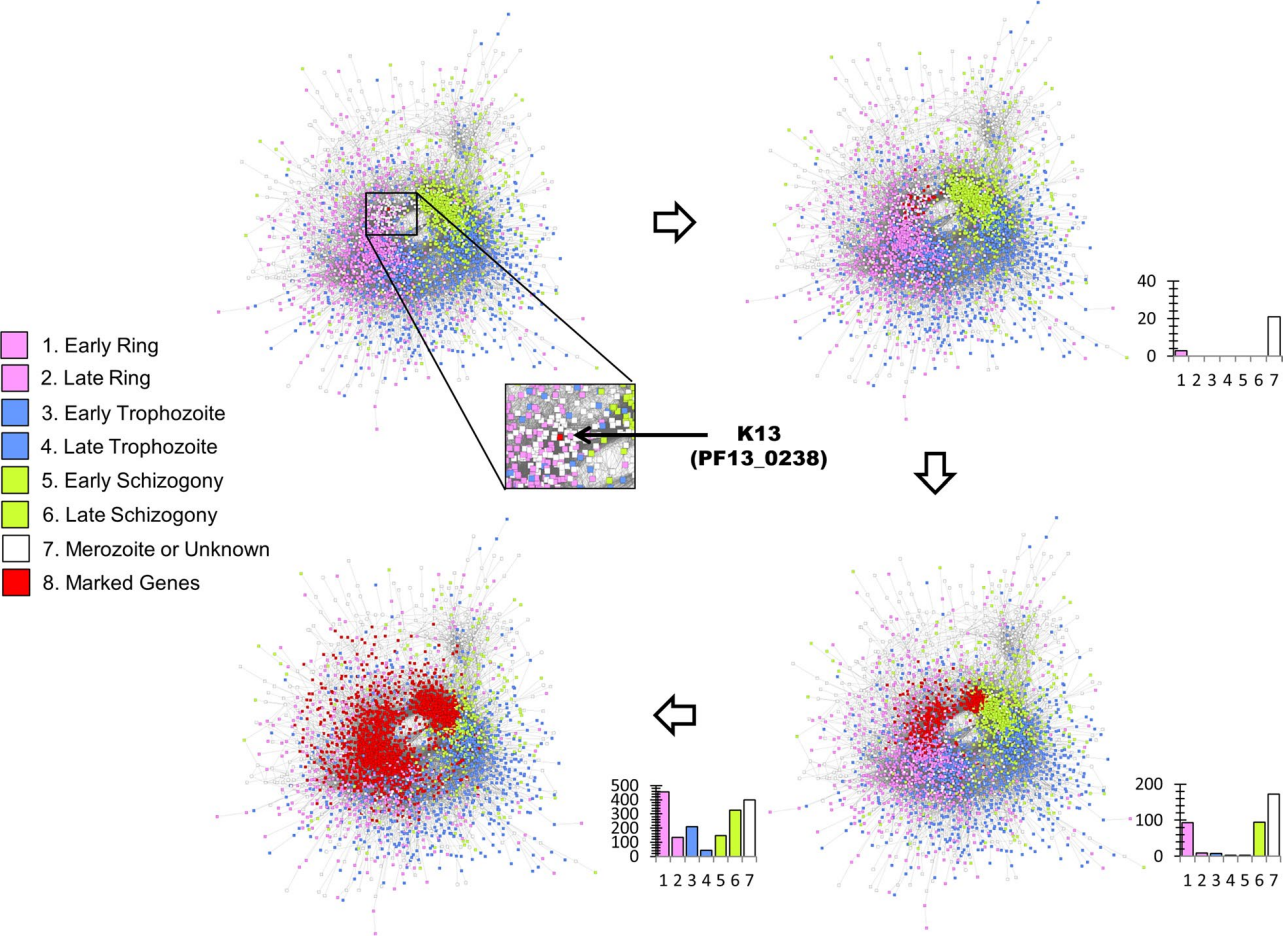
Flowchart showing the neighboring genes of the *k13* gene in co-expression based interaction network. The *P. falciparum* gene–gene interaction network based on co-expression data is recovered from STRING v10 [32]. The unconnected nodes are not considered in this analysis. The force directed layout is used to plot the network. The genes are colored according to the maximum expression stage data [34] recovered from PlasmoDB [30]. The genes highlighted in “red” color, are the first, second and third neighbor genes of the *k13* gene, shown using four networks in clockwise orientation. The neighbor genes are marked using Cytoscape v3.2.1. The barplot with each network represents the number of genes expressed in each stage per diffusion

**Fig. 3:**
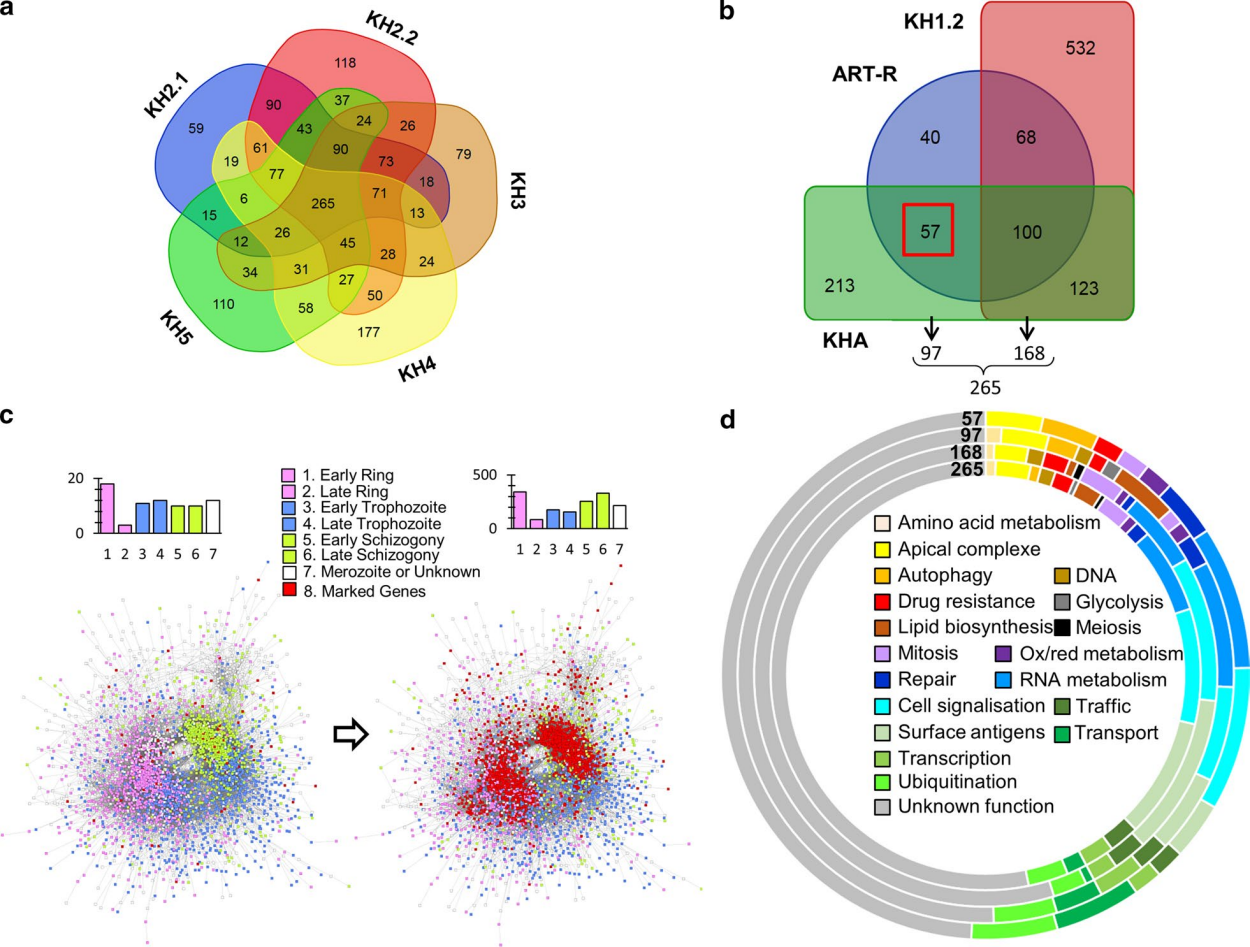
Selection of artemisinin resistance associated genes. **a** Emergence of a common genetic background associated with all five ART-R subpopulations. 5-way Venn-diagram emphasizes great importance of the 265 common set of significant genes in ART-R subpopulations. Genes have at least one significant SNP in the ART-R subpopulations. **b** Selection and diffusion of the artemisinin-resistance background after crossing with KHA parasites. Red square: genes with significant ALT alleles in the admixed population and in the ART-R subpopulations (57 genes). Alleles for these genes may have been acquired by diffusion after crossing. **c** The 97 genes (76 with connections) specific to ART-R subpopulation genetic background are connected with ring or schizont stage in *P. falciparum*. First neighboring genes (in red) in the interaction network based on co-expression data recovered from STRING v10 [32]. The unconnected nodes are not considered in this analysis. The genes are colored according to the maximum expression stage data [34]. The bar plot with each network represents the number of genes expressed in each stage per diffusion. **d** Conservation of biological pathways during selection and diffusion of significant mutations from original ART-R related genetic background

Network-based parasite stratification and K13 connections in the co-expression network establish possible relationships between populations and gene functions. Indeed, most of the genes co-expressed with K13 are expressed during the ring and schizont stages, supporting the observation that K13 regulates the transition from ring to schizont stages (Fig. 2). Biological pathways and functions in ART-R subpopulations (KH2.1, KH2.2, KH3, KH4, KH5) were investigated using significant genes, defined as genes carrying at least one significant SNP. The level of significance of SNPs was based on comparison of the ALT allele frequency in a given subpopulation with respect to ART-S ancestral subpopulation KH1.1. ART-R subpopulations have 265 significant genes in common (Fig. 3a; Additional file 10). A set of 168 significant genes was found in common with the KH1.2 ART-S subpopulation (Fig. 3b; Additional file 10). The STRING-induced sub-networks derived from the 265 and the 168 genes overlapped, suggesting the presence of a common background for all subpopulations emerging from the ancestral population KH1.1 (Additional file 11).

The whole 265 significant genes displayed GO term association with cell signaling, gene expression, post-translational regulation, RNA metabolism (tRNA and mRNA modification…) and organelle related functions (Additional file 12). The 168 genes set GO annotation revealed conserved enrichment for cell signaling, gene expression and organelle functions (Additional file 13). About 60/265 genes encode membrane proteins encompassing 19 surface antigens including *msp1* and some specific *var* genes (Additional file 14). Several genes are associated with cell signaling and a significant number of these are protein kinases (7/17). The *Pfmdr2* gene is part of the 168 genes set and is associated with the background D484I mutation, which was present in KH1.2 ART-S subpopulation.

The *ARPS10* (apicoplast ribosomal protein S10) encoding gene was found to carry two different mutations V127 M and D128H, which were significantly enriched in ART-R and admixed subpopulations, whereas KH1.2 had only D128H mutation. The V127 M mutation was previously described in *Neisseria gonorrhea* to be associated with tetracycline resistance [44]. Protein model prediction revealed that ARPS10 central domain is highly similar to the bacterial protein, and that the mutated valine is at the same place in a loop (Additional file 15). *Plasmodium falciparum* doxycycline resistance was not characterized so far in SEA, but background mutations may contribute to lower efficiency of antibiotic therapy that remains to be evaluated.

### Description of ART-R subpopulations genetic background

To assess the ART-R subpopulations background genes, the 168 genes set found in common with KH1.2 ART-S subpopulation were removed from the total of 265 genes, and the remaining genes were analysed (Fig. 3b; Additional file 10). The 97 genes were distributed in the Ring and Schizont categories of the co-expression network (Fig. 3c). An enrichment for the GO term “anion binding” is the only one which is reminiscent of cell signaling pathway in the remaining set of 97 genes (Additional file 16). Indeed, protein kinase activity is now restricted to the presence of ARK2 (Aurora Related Kinase 2), a potential drug target [45], and the CCAAT-box DNA binding protein is the only transcription factor remaining at this step. Analysis also brings out the association of the 97 genes set with specific mutations in the PDE1 phosphodiesterase, which is involved in the regulation of cGMP concentration. The *pde1* gene carries 3 mutations found in some individuals of the KH1.1 ancestral population, but only S506L was fixed in all the ART-R subpopulations (Additional file 17). Specificity of PDE1 for cGMP was characterized experimentally [46] and the role of the cyclic nucleotides in parasite division and differentiation was well established [47]. Genes in the same GO term functional group as *k13* are associated with poly-ubiquitination [*MAL7P1.19* Ubiquitin transferase (UT) and *rpn10* proteasome subunit], signal transduction (*pi3k*), autophagy (*atg7* and *atg18*) and ferredoxin (Additional file 16). Background mutations associated with the ferredoxin gene (*fd*) were found to be significant in all ART-R and admixed subpopulations. Interestingly the ferredoxin D193Y background mutation was not always associated with the *k13* mutations in the KH3 subpopulation. Ferredoxin gene is also part of a functional group of genes encoding apicoplast proteins of unknown function. The PfCRT encoding gene is also part of ART-R subpopulations background and was associated with 13 mutations including the two background mutations N326S and I356T. KH1.2 had no significant *Pfcrt* mutations. An additional R371I mutation was found in ART-R and the admixed subpopulation KHA. KH4 isolates carry one more significant mutation T256I.

### The background genes of artemisinin resistance associated pathways

Parasite population characterization suggests that emergence of *k13* mutations in Cambodia was tightly related to the presence of subpopulations like KH2, KH3, KH4 and KH5, which provides an appropriate background for acquisition of artemisinin resistance. In the admixed subpopulation KHA, 22 isolates out of the 49 KHA isolates had no *k13* allele. This high proportion may be related to the focus of the present study on the early period of emergence of artemisinin resistance (2008–2011). Out of the 5 background genes mentioned above, the *fd* gene was the only significant gene in the KHA subpopulation when compared to the total population of 167 isolates. When KHA was compared with the ancestral population KH1.1 as reference, the number of significant genes increased to 467. These KHA specific gene list overlaps with that of ART-R 97 genes set. The presence of overlapping functions and genes (Fig. 3d) supports the idea that KHA genetic background in the period 2008–2011 was acquired through crossing of ART-R parasites with various populations either ancestral, resistant or a pre-existing admixed population as suggested by the comparison of the stratification approaches used in this study (Fig. 1; Additional file 9). The artemisinin resistance genetic background was defined as the intersection between ART-R 97 subpopulation background genes and the 467 KHA significant genes (Fig. 3b; Additional file 18), and a final list of 57 genes was recovered (Additional file 10).

The 57 genes set is supported by 213/230 mutations that were significant in KHA and at least one of the resistant subpopulation (Additional file 7). Diffusion of the ART-R subpopulations genetic background is also demonstrated by the fact that parasites having one of the *k13* resistant allele present the higher proportion of these mutations (Additional file 19). Genes had a median of 3 mutations with a maximum of 27 mutations in PFL0940c of the *var* genes family (Additional file 20). High number of mutations in some samples from specific subpopulation was in agreement with the observation that some KHA isolates show different classification between hierarchical clustering and network based stratification (Fig. 1; Additional file 9).

**Fig. 4:**
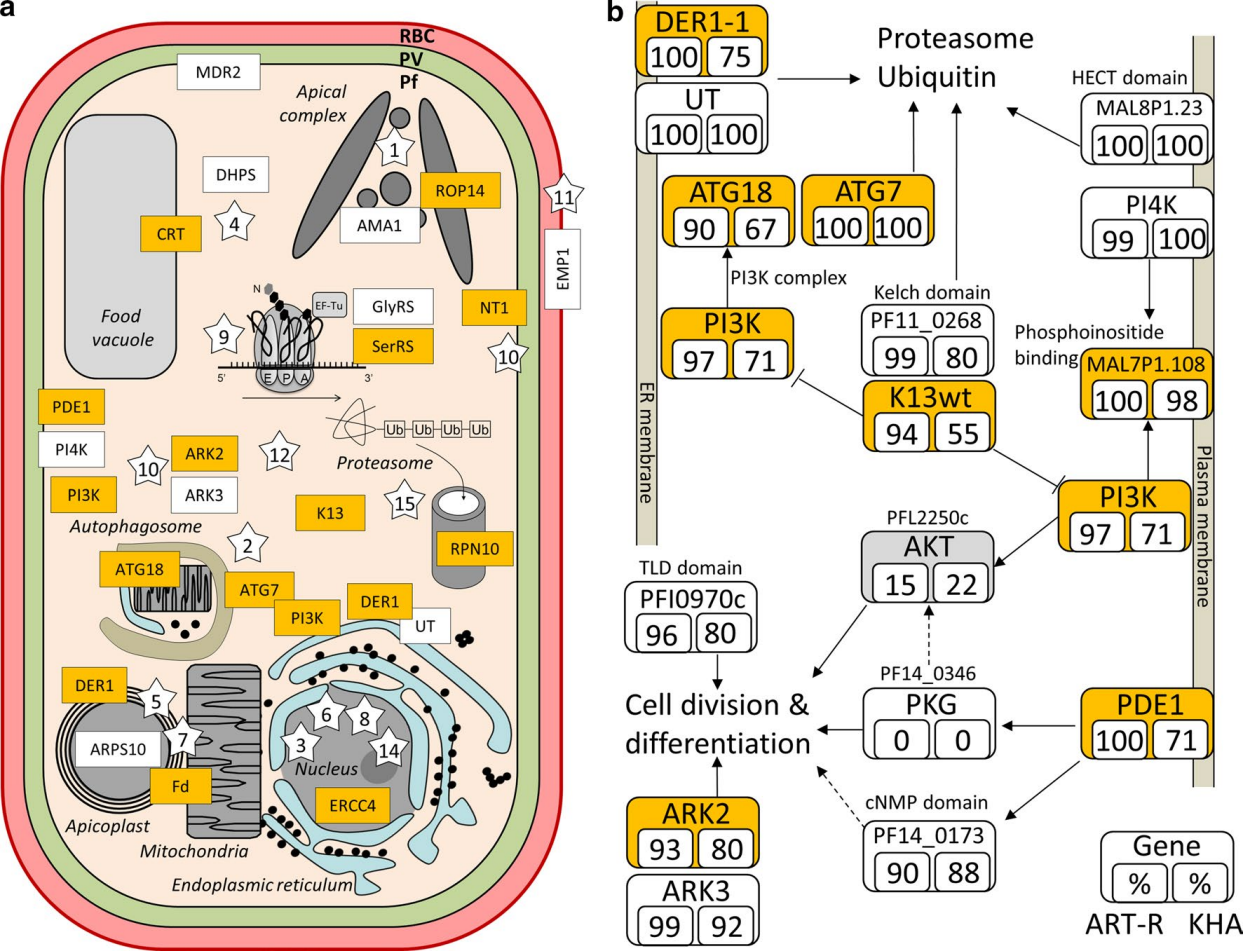
Major functions or pathways related with the genetic background of artemisinin resistant Cambodian parasites. **a** Cell localization of pathways (stars) and significant genes (boxes) with major known biological functions. Annotation terms were taken from genes current description in PlasmoDB [30] and GO terms. Star numbers refer to biological pathways or cell localization: 1, Apical complex; 2, Autophagy; 3, DNA/repair; 4, Drug resistance; 5, Lipid biosynthesis; 6, Mitosis/Meiosis; 7, Oxidation–reduction; 8, RNA metabolism (mRNA); 9, RNA metabolism (tRNA); 10, Cell signalization; 11, Surface antigen; 12, Traffic; 13, Transport; 14, Transcription; 15, Ubiquitination. **b** Interplay between ubiquitination and cell division in artemisinin resistant parasites. Frequency of isolates having at least one significant mutation or a rare mutation was calculated for the ART-R subpopulations and KHA parasites. Plasma membrane and endoplasmic reticulum membrane are represented by grey bars. Schema was built from protein–protein interactions or relationship given in databases and literature. Orange boxes: genes from the 57 genes set; White boxes: significant genes from the 265 set. AKT has grey box because it contains no significant mutations, but only rare mutations

**Fig. 5:**
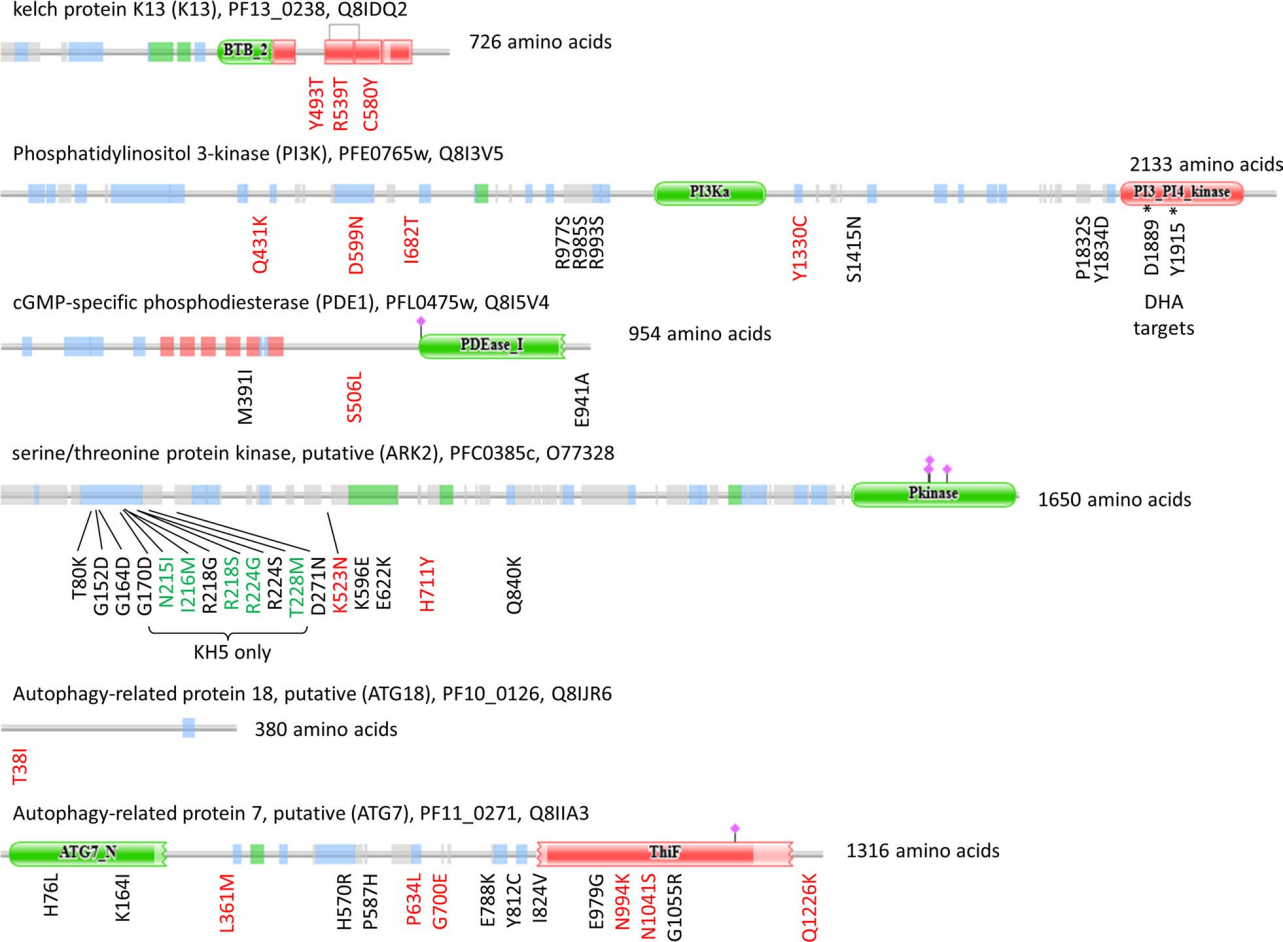
Distribution of the significant non-synonymous SNPs along amino acid sequence of K13, PI3K, PDE1, ARK2, ATG18 and ATG7 proteins. Domain structure of Kelch protein (K13), phosphatidylinositol 3-kinase (PI3K), cGMP-specific phosphodiesterase (PDE1), serine/threonine protein kinase (ARK2), autophagy related protein (Atg18 and Atg7), recovered from Pfam database server. Amino acid substitution and position in the protein are given for all non-synonymous mutations. Color code of mutations: black, not significant in any subpopulation; green, significant in only one subpopulation; red, significant mutations observed in several subpopulations

The cell signaling enzymes ARK2, PI3K and PDE1 found in the 97 genes set were conserved in the 57 genes set (Fig. 4a), but artemisinin resistance emerged now as a complex interplay between these genes to regulate ubiquitination and cell division or differentiation (Fig. 4b). Polyubiquitination is the major pathway degrading most cellular proteins in eukaryotic cells. Furthermore, artemisinin could lead to mitophagy after targeting the mitochondria [48]. Autophagy is represented in the 57 genes set with mutations in genes encoding ATG18 and ATG7 (Additional file 10). ATG18 has a single T38I mutation (Fig. 5) which may correspond to a phosphorylation site as it is reminiscent of the threonine at position 56 in the yeast protein which was phosphorylated in nutrient rich conditions [49]. Significant mutations in ATG7 are dispersed and are surrounding the E1-specific ThiF domain (Fig. 5). Autophagy pathway is downstream of PI3K and K13. PI3K is connected to ATG18 as a member of pre-autophagosomal structure (PAS) which is recruited by ATG9 at PI3P rich regions of the ER membrane [50]. Autophagy is a response of eukaryotic cells to oxidative stress and is activated during cell cycle arrest to reduce metabolic activity [51–53], two important features of the artemisinin resistance phenotype. Increase of autophagy activity in ART-R parasites could also negatively regulate cell death [54, 55].

## Discussion

### Interplay between ubiquitination and cell signalization pathways

Relationship between protein turn-over at the proteasome and artemisinin resistance is supported by missense mutations in K13. K13 is expected to work as part of an E3-like protein complex interacting with the target proteins through the Kelch domain. Poly-ubiquitination of PI3K kinase mediated by K13 was described experimentally [22], but the E3 ubiquitin ligase containing K13 may also target many more proteins. Ubiquitin binds to lysine, cysteine, serine or threonine residues or at the N-terminus on the protein substrate. The final 57 genes set presents 95 missense mutations concerning such residues over the 230 mutations that are significant in at least one subpopulation. More information will be needed to identify those who are indeed targeted by the K13-associated E3-complex. The S102C mutation found in the *P. falciparum* derlin protein (DER1-1) is intriguing, as it is the only significant mutation observed in this gene. Derlin1 is involved in polyubiquitination of ER-located proteins and was also found to be located at the apicoplast in *P. falciparum*. The K523 N mutation is observed in ARK2 (Aurora Related Kinase 2), which was fixed in the KHA subpopulation. ARK2 is a serine/threonine protein kinase and is essential for asexual parasite development [56]. Mutations in *Pfark2* are all clustered in two regions of about 200 amino acids in the N-terminus part of the protein (Fig. 5). Most of these mutations globally increase the level of hydrophobicity of the ARK2 N-terminus domain of the protein suggesting that these two regions are probably part of a protein interface.

ATG18, DER1-1 and PDE1 are the three proteins out of the 12 with a single mutation from the 57 genes set, where a serine or a threonine is lost by mutation. The S506 PDE1 residue could modulate phosphodiesterase activity regulating cGMP concentration and activity of the single cGMP dependent protein kinase PKG found in *P. falciparum* (PF14_0346). Additionally, no connections were found with transcription factors. The two ApiAP2 protein which were in 265 genes set were absent in the 97 genes set. The SET4 and CAAT-binding proteins were not conserved in the final 57 genes set. Other approaches must be considered to look for possible inference of the mammals KEAP1/NRF2 model in the *P. falciparum* artemisinin resistance mechanism [57].

### Fixation of mutations in the ART-R subpopulations

Genetic background of subpopulations suggests that resistance to artemisinin appears as a selection process among parasites from subpopulations. In the case of *k13*, different subpopulations carry different alleles: Y493H, R539T or C580Y. The gene is unique among all ART-R related genes because it is the only one for which mutually exclusive mutations are found. The *k13* gene was found to be significant in donor populations where only one *k13* mutation was present (*p* = 3.92E^−11^), but also in KH5 (*p* = 1.0296E^−4^) where all the 3 different exclusive mutations were present. KH5 is a donor for R539T mutation, as it is absent in other subpopulations except KHA. The fixation of *k13* mutations was not so different from the other genes of the ART-R subpopulations genetic background. There are 524 mutations occurring in the 97 genes that were significant in at least one ART-R subpopulation (Additional file 7). None of the *k13* mutations were found in the KH1.1 ancestral population, and this was also true for several mutations related to the 97 genes set (154/524). These 154 mutations suggest that ART-R subpopulations specific mutations that were not found in the KH1.1 samples were certainly rare in the ancestral population, including *k13*. The genetic drift during the emergence of subpopulations and the selection performed afterwards is also characterized by the presence of only 57/524 significant mutations that are found in all the ART-R subpopulations, but these mutations are located in 46/97 genes of the ART-R subpopulations genetic background confirming that selection process may have targeted specific pathways. Altogether, these results suggest that selection of the artemisinin genetic background happened in the daughter subpopulations and not in the ancestral population.

### Role of KHA parasites in the diffusion of resistance to artemisinin

The process of emergence of artemisinin resistance could be summarized with emergence-selection-diffusion model (ESD). This model is similar to the population shifting balance theory described by Wright [58]. The present work describes the emergence of common genetic background in different subpopulations. Parasites among these subpopulations presented a large number of fixed mutations compared to the ancestral population KH1.1. Genetic drift in small populations and relationship with resistance were described previously [4, 11–13]. Emergence of *k13* resistant alleles was only observed in the genetic background of these subpopulations and alleles were clearly associated with specific subpopulations. This mechanism has now been confirmed worldwide [10].

Presence of ART-R subpopulation specific mutations associated with known drug resistance markers such as *Pfcrt* N326S and I356T background mutations or R371I found in Dd2 (in a context where K76T is present in 34/167 samples including KH1.1 samples), S436A and K540 N in *Pfdhps* and T484I background mutation in *Pfmdr2* (in combination with a new G299D mutation) further support the idea that artemisinin resistant parasites were selected among subpopulations in a context where fixation of mutations could be speed up by drug pressure [4, 59–62]. In the same way, the results suggest that background mutations in *Pfarps10* are possibly associated with doxycycline resistance even though there is little evidence of *P. falciparum* resistance to this antibiotic in Cambodia. Doxycycline resistance in *P. falciparum* can be explained in part by *pfmdt* and *pftetQ* copy number (*PFE0825w* and *PFL1710c, respectively*) and polymorphisms on *pftetQ,* which encode transporters [63]. None of these two genes show any variation in the 167 isolates, but copy number was not evaluated. The fixation of the *arps10* mutations may result from the use of doxycycline against *P. falciparum* in combination with quinine, or in anti-malarial prophylaxis [63]. It should be considered that *Pfarps10* background mutations are a side effect of the wide use of tetracycline derivatives in Cambodia, especially in the suspicion of leptospirosis epidemics [64]. The *Pfarps10* mutation contributes little to tetracycline resistance in *Neisseria gonorrhoea* alone, but was responsible for high resistance in combination with efflux pumps [63]. It can be imagined that high resistant *P. falciparum* parasites can emerge by crossing between KHA samples with Africans isolates, where high copy numbers of transporters were described [63].

## Conclusions

The present study considers an Emergence-Selection-Diffusion model (ESD) for description of biological information related to mutations accumulating in parasites during the early time period of emergence of artemisinin resistance (2008–2011). This descriptive study showed that random drift in subpopulations may help the emergence of mutations in specific biological pathways. The 168 genes set was associated with cell signaling, gene expression, organelle functions and surface antigens such as *msp1* and some *var* genes. Analysis allow the detection of common features between resistant subpopulations that are result from the selection of drug resistant parasites. The 57 background genes found in the present study, associated with the selection of *k13* mutants, are encoding proteins involved in ubiquitination, autophagy and cell signaling enzymes such as ARK2, PI3K and PDE1 which are involved in cell division and differentiation. Relationship with other malaria drug resistance markers was also present in the subpopulations. Possible resistance to tetracycline emphasize that the population structure in the GMS is promoting rapid emergence of drug resistance as hypothesized by the population shifting balance or metapopulation theories.

Diffusion of the *k13* alleles related to the early time of emergence of artemisinin resistance in Cambodia have still not diffused outside of the country, at least up to 2016 when the last large survey was performed [5, 10]. Nevertheless, the present study emphasizes the important role of KHA parasites in diffusion of artemisinin resistance in Cambodia, which are more prone to crossing with other parasites according to the ESD model and similar population genetic theories. The fear of diffusion of *k13* alleles outside GMS is real now, as some are fixed in KHA parasites, but this will need to be confirmed experimentally. The functional analysis of genomic data also suggests that biological functions associated with the 57 background genes could either be the result of environmental pressure or intrinsic consequence of genetic drift in parasites with low fitness. Several resistant markers to known drugs were found in KHA samples. This population may play a central role facilitating emergence of resistant parasites under the pressure of anti-malaria drugs. The present study of the early emerging artemisinin resistant populations suggest that newly emerging parasites are susceptible to be resistant to several anti-malarial drugs. The fitness of such parasites is under question, but tracking the diffusion of these new genetic background outside the GMS should certainly be extend to additional markers than the *k13* gene alone.

## Additional files

**Additional file 1.** List of all the isolates used for the analysis. This list provides the index of the sample, ENA accession number, locality, year of data collection, Sample ID, Study ID and the name of the file to be downloaded from the ENA database server. This list is sorted in alphabetic order of localities in Cambodia. This publication uses data and the metainformation from the MalariaGEN *Plasmodium falciparum* Community Project as described in *Genomic epidemiology of artemisinin resistant malaria.*

**Additional file 2.** Variant (SNP) Calling Pipeline. This flowchart describes the major steps of the pipeline to call significant SNPs for the population study. The numbers on the left of the flowchart are the number of SNPs kept at each step. The detailed steps to select relevant SNPs are mentioned on the right side of the flowchart.

**Additional file 3.** Mapping quality of the 167 samples. The Mapping quality (MQ: Root-mean-square mapping quality of covering reads) values (x-axis) averaged over 167 isolates for each SNP (y-axis) plotted along the genome. The average quality value for each SNP is calculated as the ∑values in different isolates/number of isolates having that SNP. The red dotted vertical lines represent the last SNP in each chromosome and the blue dotted horizontal line represents the MQ value 29. **a** Average MQ values of all the unique SNPs along the full genome. **b** Average MQ values of all the unique SNPs in the coding core. SNPs in 100 kb region at the starting and end of all the 14 chromosomes removed.

**Additional file 4.** The density of SNPs for DA (∑non-REF alleles/∑DP4) averaged over 167 isolates for each SNP. This figure represents the density of SNPs with a density function fitted on the histogram (red line) and the minima of the curve after DA ≥ 0.5 (dotted blue line). All the SNPs above the threshold DA ≥ 0.7 were included in the analyses.

**Additional file 5.** Frequency of 247,783 SNPs for non-reference allele frequency (NRAF) score and minor allele frequency (MAF) score. **a** Shows the frequency of SNPs for NRAF score. The small zoomed version of the histogram shows the NRAF values 1, 2, 165, 166 and 167 which were not include in the analyses (marked with red triangles). **b** Shows the frequency of SNPs for MAF scores. The SNPs with the MAF value below the threshold of 0.01796 (blue dotted line) correspond to the NRAF values which were not included in the analyses.

**Additional file 6.** Criteria and statistics of SNPs with uncertain ALT alleles in the isolates. Uncertain SNPs are defined as the SNPs with more than one ALT allele in at least one of the 167 isolates. **a** Shows the histogram of uncertain ALT allele frequency. All the SNPs with uncertain ALT allele frequency greater than 40% (dotted blue line at 0.4) were removed from the analyses. **b** Represents a schematic diagram of different cases considered for SNPs with uncertainties and the decision of substitution taken. Uncertainties were substituted with the most frequent ALT value or REF value in around 17% of the SNPs at this step. **c** Represents the pie chart with percent of certain SNPs, substituted SNPs and removed SNPs.

**Additional file 7.** Database presenting the list of 21,257 non-synonymous SNPs found in the 167 Cambodian isolates. Calling of SNPs is described in the “Methods” section. See full legend in the corresponding excel sheet.

**Additional file 8.** Classification of 167 samples into artemisinin resistant and sensitive Cambodian subpopulations. **a** Dendrogram representing the classification of 167 parasite isolates into 8 clusters (k = 8). The pairwise distance between two samples is calculated as the proportion of base substitution between them over the genome. Ward’s minimum variance method is used as the metric to build the dendrogram. Different clusters (subpopulations) are represented with different colors. **b** The barplot represents the number of isolates in each subpopulation described. “Green” represents ART-S isolates. “Red”, “Yellow” and “Blue” color represents isolates with C580Y, Y493H and R539T *k13* mutations, respectively. **c** The barplot represents the number of isolates in each Cambodian locality (Ratanakiri, Pursat, Tasanh and Pailin) and the colors represent the type of *k13* mutation present. **d** The barplot represents the number of isolates in each locality and the colors represent the associated subpopulation to each isolate.

**Additional file 9.** Hierarchical clustering of 167 isolates based on the network based stratification method [27]. Interaction network was recovered from STRINGv10 and interaction evidence from all the sources was used. Only top 10% of the interactions were included in the analysis. Similarity matrix was computed using consensus clustering, which was performed by selecting 80% mutated genes and 80% isolates 100 times randomly and iterating NMF clustering 10 times. This similarity matrix was then used to build a dendrogram for all 167 isolates using Euclidean distance matrix and ward minimum variance method in R. Colors for different clusters were assigned by comparison with the hierarchical clustering result based on 21,257 SNPs. The isolates classified in different clusters in two approaches (SNP based and Network based) are pointed with triangles. The colors of the triangles correspond to the hierarchical classification.

**Additional file 10.** List of all the significant genes specific to ART-R subpopulations genetic background (265 genes). These genes have at least one significant SNP in KH2.1, KH2.2, KH3, KH4 and KH5 subpopulations. The 168 genes set are genes significant in ART-S subpopulation KH1.2 and all the ART-R subpopulations. The complementary set of 97 significant genes is specific to the ART-R subpopulations and are not in the ART-S subpopulation KH1.2 background. The 57 genes have at least one significant SNP in all the ART-R subpopulations, in the admixed subpopulation KHA and no significant SNP in ART-S subpopulation KH1.2. Annotation terms describing the function, the pathway or cell localization of the protein product were taken from genes current description in PlasmoDB and GO terms related to the genes or Pfam domains.

**Additional file 11.** Network representation of overlapping gene sets associated with ART-R subpopulations (set of 265 genes) and the ART-S subpopulation KH1.2 common resistance background (set of 168 genes). The networks for the two gene sets based on co-expression data are recovered from STRING v10. The edges connecting the genes, have the co-expression evidence score greater than 0.5. The nodes and edges in “red” color represent the interaction network based on coexpression for ART-R subpopulations gene set. The nodes and edges in “white” color represent the interaction network based on coexpression for ART-S sunpopulation KH1.2 common resistance background genes set. For the ART-R subpopulation specific genes set, out of the 265 genes only 173 genes are used for overlap and for the KH1.2 common resistance background genes set, out of 168 only 113 genes are used for overlap. Other genes (nodes) are removed either because of no interactions (before/after overlap) or STRING confidence score below 0.5. The representation of overlapping coexpression network is done in Cytoscape v3.2.1 using the DyNet Analyzer plugin.

**Additional file 12.** The functionally grouped networks of GO terms and pathways for ART-R subpopulation specific genes set (265 genes). This network represents the associations between the GO terms based on the similarity of the genes. The nodes represent the GO terms and the edges are the associations based on kappa score, which is also used for defining functional groups. Each functional group is represented with the most significant GO term in the functional group. The “Triangles” represent the metabolic pathways, “Ellipse” represents the GO terms associated to biological processes, “Hexagon” represents the GO terms associated to cellular component and the “Rectangles” represent the GO terms associated to Molecular functions. Different colors signify different GO terms functional groups. Nodes with more than one color represents the GO terms included in more than one functional group. The network of GO terms functional group is built in Cytoscape v3.2.1 using the plugin ClueGO v2.2.4.

**Additional file 13.** The functionally grouped networks of GO terms and pathways for ART-S subpopulation KH1.2 common resistance background genes set (168 genes). This network represents the associations between the GO terms based on the similarity of the genes. The nodes represent the GO terms and the edges are the associations based on kappa score, which is also used for defining functional groups. Each functional group is represented with the most significant GO term in the functional group. The “Triangles” represent the metabolic pathways, “Ellipse” represents the GO terms associated to biological processes, “Hexagon” represents the GO terms associated to cellular component and the “Rectangles” represent the GO terms associated to Molecular functions. Different colors signify different GO terms functional groups. Nodes with more than one color represents the GO terms included in more than one functional group. The network of GO terms functional group is built in Cytoscape v3.2.1 using the plugin ClueGO v2.2.4.

**Additional file 14.** The functionally grouped networks of GO terms and pathways with associated genes for ART-S subpopulation KH1.2 common resistance background genes set (168 genes). This network represents the associations between the GO terms based on the similarity of the genes. The nodes represent the GO terms and the edges are the associations based on kappa score, which is also used for defining functional groups. Each functional group is represented with the most significant GO term in the functional group. The “Triangles” represent the metabolic pathways, “Ellipse” represents the GO terms associated to biological processes, “Hexagon” represents the GO terms associated to cellular component and the “Rectangles” represent the GO terms associated to Molecular functions. Different colors signify different GO terms functional groups. Nodes with more than one color represents the GO terms included in more than one functional group. The network of GO terms functional group with associated genes is built in Cytoscape v3.2.1 using the plugins ClueGO v 2.2.4 and CluePedia v 1.2.4.

**Additional file 15.** Structural comparison of 30S ribosomal protein S10 protein models from *Neisseria gonorrhoeae*, *Neisseria meningitidis* and *Plasmodium falciparum*, which can mediate tetracycline resistance. **a** Model of the *N. gonorrhoeae* 30S ribosomal S10 protein (NCBI GI#501495768), colored in blue. **b** Model of the *N. meningitidis* 30S ribosomal S10 protein (NCBI GI#488148952), in green. **c** Model of the *P. falciparum* PF3D7_1460900.1 protein, with the central domain colored in red, with two N-terminal (residues 1–68) and C-terminal (residues 180–274) domains colored grey. **d** Model of the *P. falciparum* PF3D7_1460900.2 protein, with the central domain colored in forest green, with two N-terminal (residues 1–68) and C-terminal (residues 176-268) domains colored grey. **e** Shows the superposition of **a** (blue) and **c** (red), with a TM-score of 0.8549. **f** Shows the superposition of **a** (blue) and **d** (forest green), with a TM-score of 0.8736. **g** Shows the superposition of **b** (green) and **c** (red), with a TM-score of 0.8610. **h** Shows the superposition of **b** (green) and **d** (forest green), with a TM-score of 0.8786. Proteins models were constructed using the RaptorX server [40]. TM-score and protein superposition determined using TM-align [43]. TM-scores above 0.5 indicate the proteins have the same fold. All images were rendered in PyMOL. A zoom in the loop containing the putative tetracycline resistance mutation is inserted in **e**–**h** panels.

**Additional file 16.** The functionally grouped networks of GO terms and pathways with associated genes for ART-R subpopulations specific genes set (97 genes). This network represents the associations between the GO terms based on the similarity of the genes. The nodes represent the GO terms and the edges are the associations based on kappa score, which is also used for defining functional groups. Each functional group is represented with the most significant GO term in the functional group. The “Triangles” represent the metabolic pathways, “Ellipse” represents the GO terms associated to biological processes, “Hexagon” represents the GO terms associated to cellular component and the “Rectangles” represent the GO terms associated to Molecular functions. Different colors signify different GO terms functional groups. Nodes with more than one color represents the GO terms included in more than one functional group. Different parts of the network are marked with general annotation terms. The region with genes closely associated to *k13* gene (*PF13_0238*) based on GO terms are marked with a circle. Genes are also associated to autophagy (PF11_0271 and PF10_0126). The network of GO terms functional group with associated genes is built in Cytoscape v3.2.1 using the plugins ClueGO v 2.2.4 and CluePedia v 1.2.4.

**Additional file 17.** Distribution of significant mutation in PDE1 protein of *P. falciparum* in described Cambodian subpopulations. Protein description was generated using Pfam database web server. Amino acid substitution and position in the protein are given for the three mutations.

**Additional file 18.** The functionally grouped networks of GO terms and pathways with associated genes for artemisinin resistance background genes set (57 genes), obtained by overlapping KHA specific genes (467 genes) and ART-R subpopulations specific genes (97 genes). This network represents the associations between the GO terms based on the similarity of the genes. The nodes represent the GO terms and the edges are the associations based on kappa score, which is also used for defining functional groups. Each functional group is represented with the most significant GO term in the functional group. The “Triangles” represent the metabolic pathways, “Ellipse” represents the GO terms associated to biological processes, “Hexagon” represents the GO terms associated to cellular component and the “Rectangles” represent the GO terms associated to Molecular functions. Different colors signify different GO terms functional groups. Nodes with more than one color represents the GO terms included in more than one functional group. The network of GO terms functional group is built in Cytoscape v3.2.1 using the plugin ClueGO v2.2.4.

**Additional file 19.** Relationship between *k13* alleles and the number of significant mutations in the 57 genes occurring by diffusion in the KHA isolates according to ESD model. A set of 213 mutations were found in common between KHA significant mutations and in at least one ATR-R subpopulation. Horizontal axis corresponds to the number of mutations among the 213 mutations set found in one isolate. Vertical axis refers to the *k13* alleles. Matrix gives the number of isolates with corresponding genetic features. Color code refers to *k13* alleles: green, wildtype; red, C580Y; blue, R539T; yellow, Y493H.

**Additional file 20.** Distribution of the 213 significant mutations supporting the 57 background genes. These mutations are found in common between KHA significant mutations and those found in at least one ATR-R subpopulation. They are suspected to be present in the KHA subpopulation after diffusion, by crossing with parasites from the ART-R subpopulations.
