# Supplementary figures and images for: Functional analysis of Plasmodium falciparum subpopulations associated with artemisinin resistance in Cambodia
Source: Malar J. 2017 Dec 19;16:493. doi: 10.1186/s12936-017-2140-1 (PMC5735551; doi:10.1186/s12936-017-2140-1)

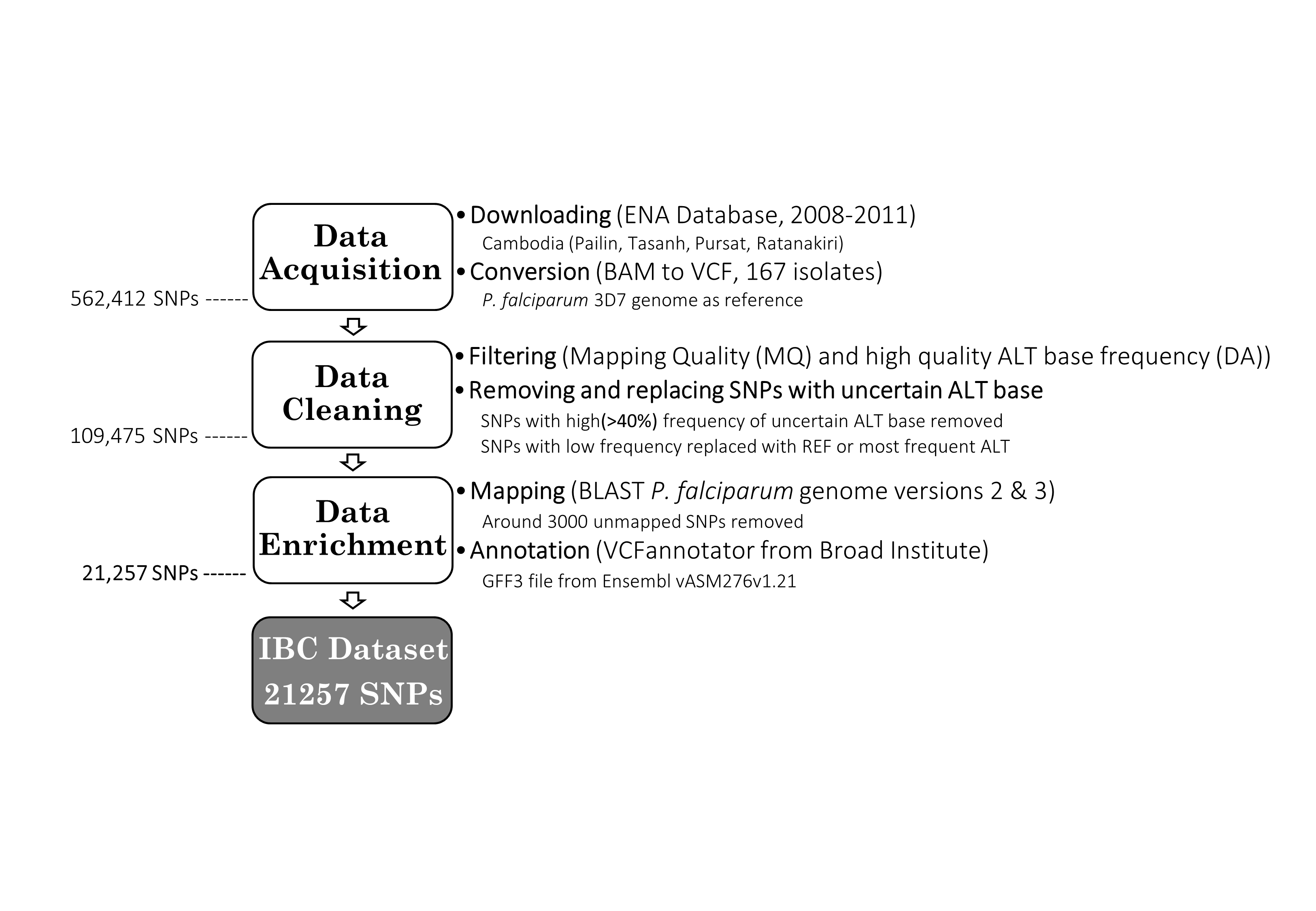

Supplement: Supplementary file 2 — Additional file 2. Variant (SNP) Calling Pipeline. This flowchart describes the major steps of the pipeline to call significant SNPs for the population study. The numbers on the left of the flowchart are the number of SNPs kept at each step. The detailed steps to select relevant SNPs are mentioned on the right side of the flowchart. [file 12936_2017_2140_MOESM2_ESM.tif]

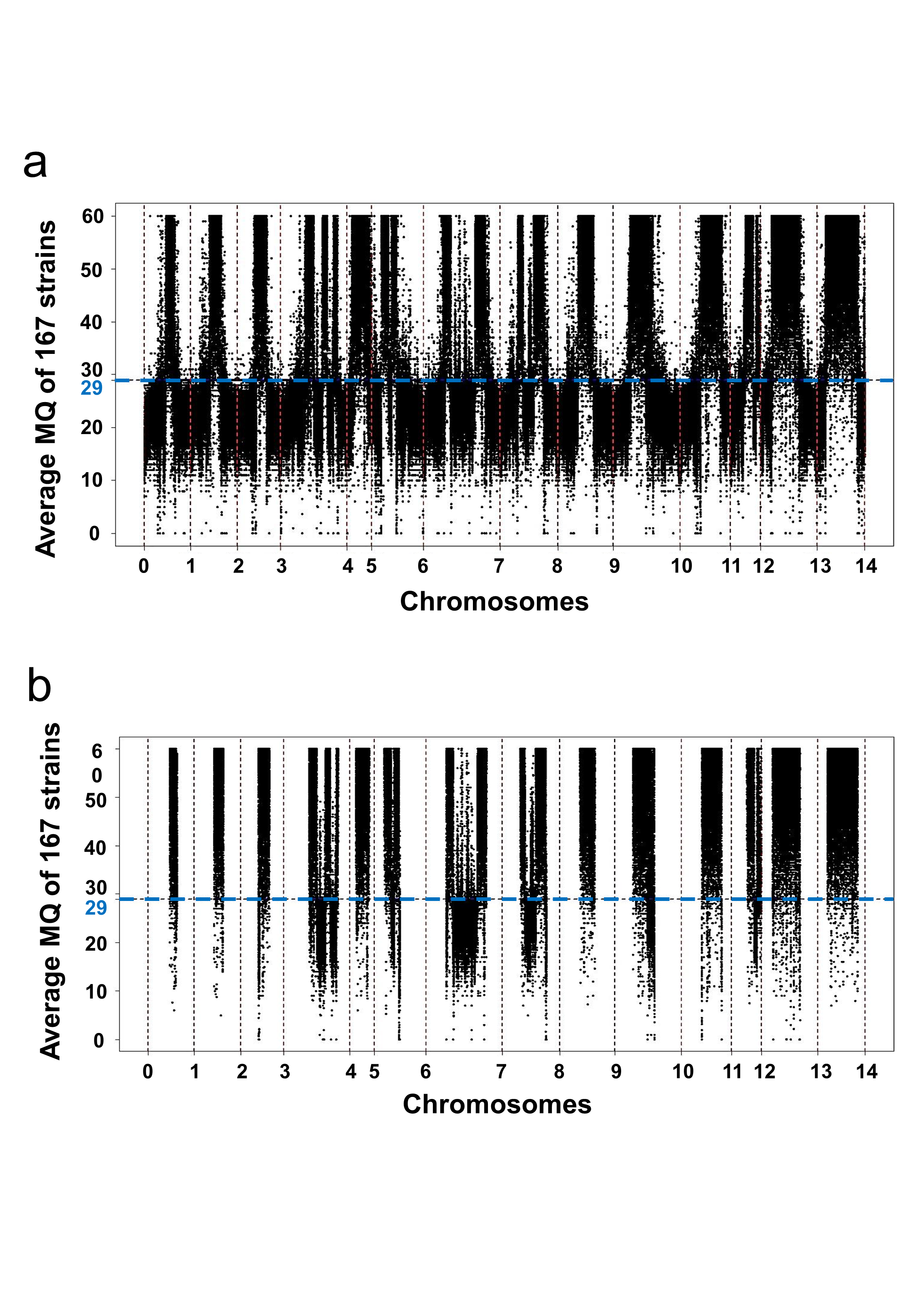

Supplement: Supplementary file 3 — Additional file 3. Mapping quality of the 167 samples. The Mapping quality (MQ: Root-mean-square mapping quality of covering reads) values (x-axis) averaged over 167 isolates for each SNP (y-axis) plotted along the genome. The average quality value for each SNP is calculated as the ∑values in different isolates/number of isolates having that SNP. The red dotted vertical lines represent the last SNP in each chromosome and the blue dotted horizontal line represents the MQ value 29. a Average MQ values of all the unique SNPs along the full genome. b Average MQ values of all the unique SNPs in the coding core. SNPs in 100 kb region at the starting and end of all the 14 chromosomes removed. [file 12936_2017_2140_MOESM3_ESM.tif]

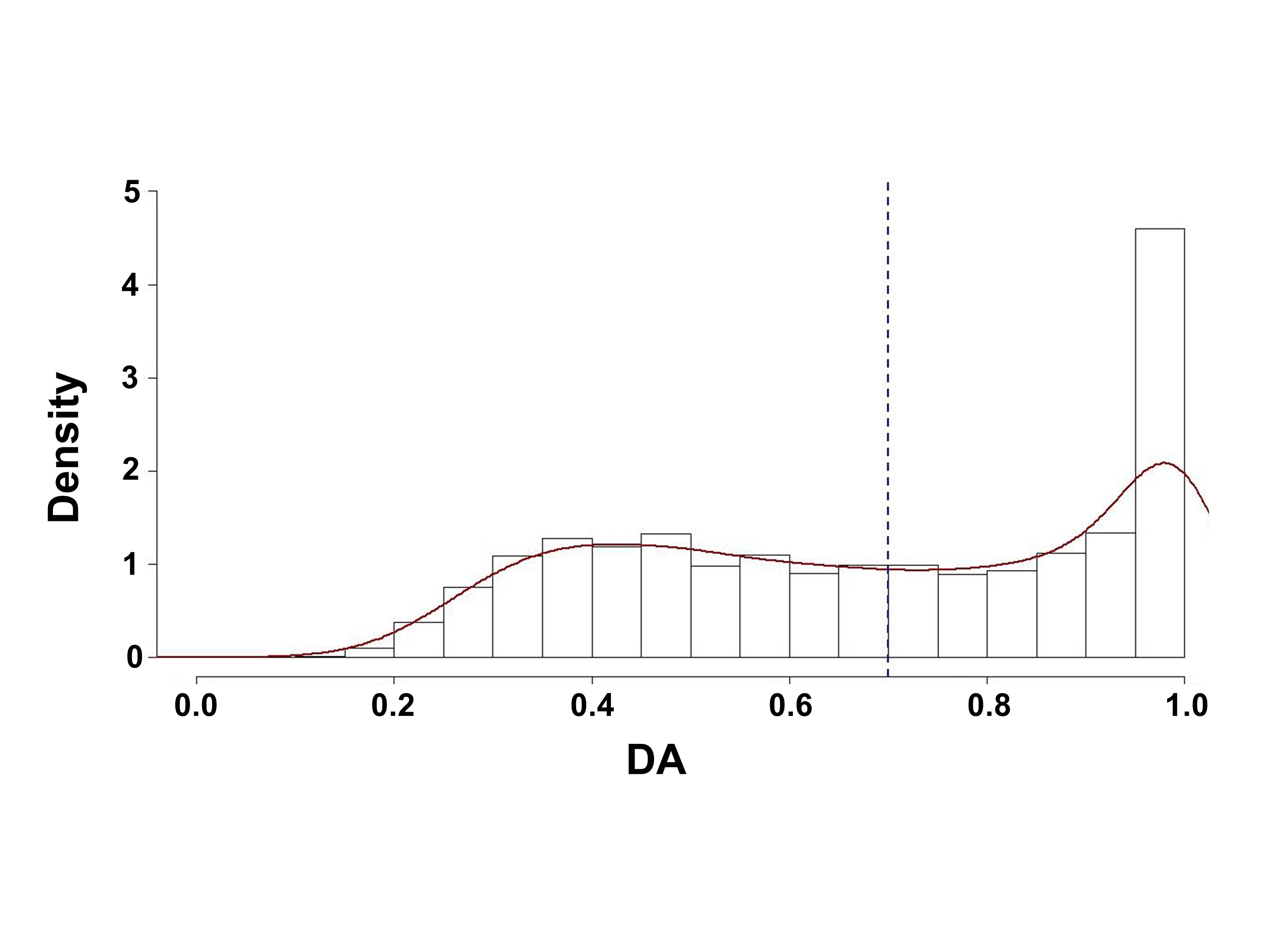

Supplement: Supplementary file 4 — Additional file 4. The density of SNPs for DA (∑non-REF alleles/∑DP4) averaged over 167 isolates for each SNP. This figure represents the density of SNPs with a density function fitted on the histogram (red line) and the minima of the curve after DA ≥ 0.5 (dotted blue line). All the SNPs above the threshold DA ≥ 0.7 were included in the analyses. [file 12936_2017_2140_MOESM4_ESM.tif]

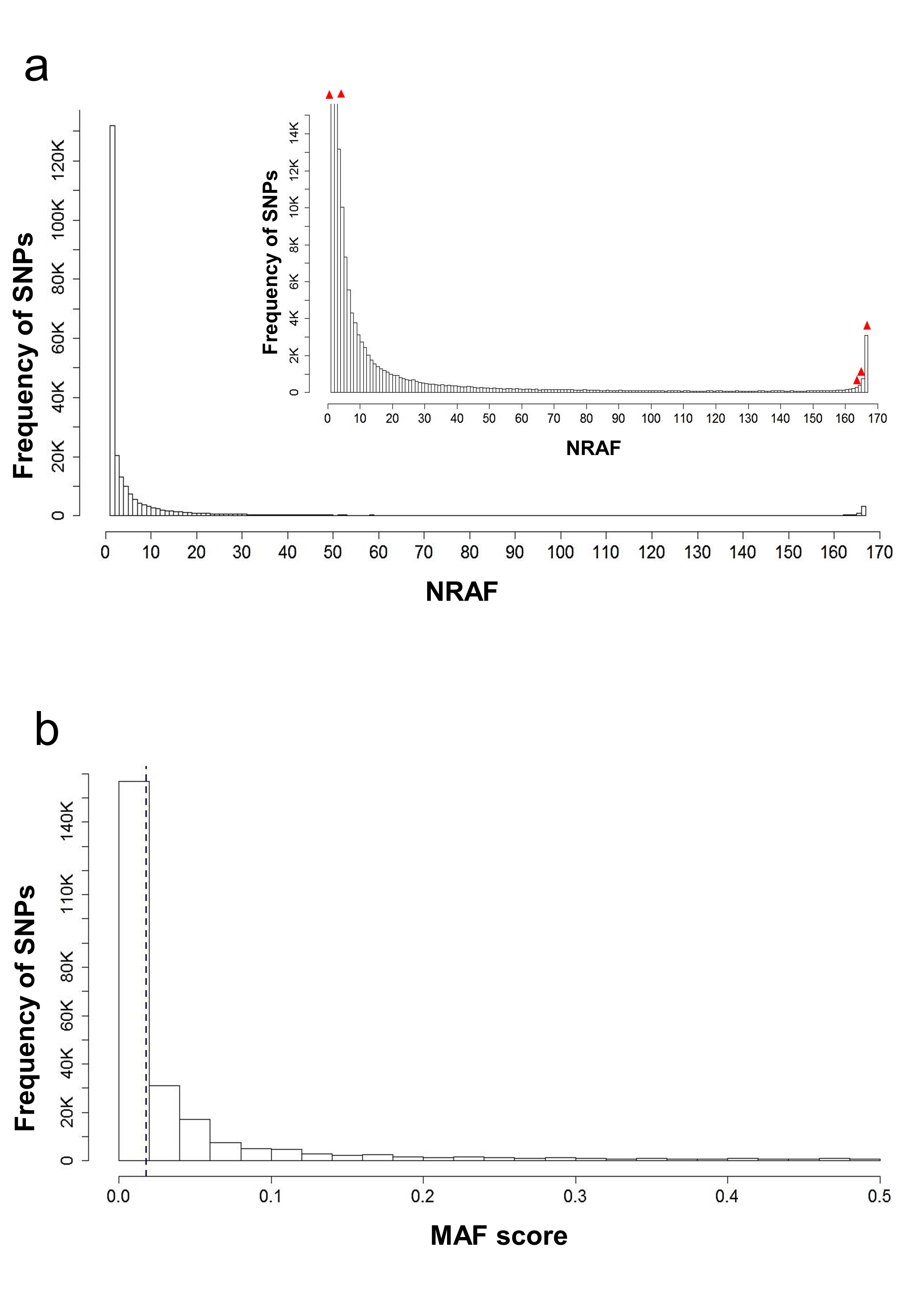

Supplement: Supplementary file 5 — Additional file 5. Frequency of 247,783 SNPs for non-reference allele frequency (NRAF) score and minor allele frequency (MAF) score. a Shows the frequency of SNPs for NRAF score. The small zoomed version of the histogram shows the NRAF values 1, 2, 165, 166 and 167 which were not include in the analyses (marked with red triangles). b Shows the frequency of SNPs for MAF scores. The SNPs with the MAF value below the threshold of 0.01796 (blue dotted line) correspond to the NRAF values which were not included in the analyses. [file 12936_2017_2140_MOESM5_ESM.tif]

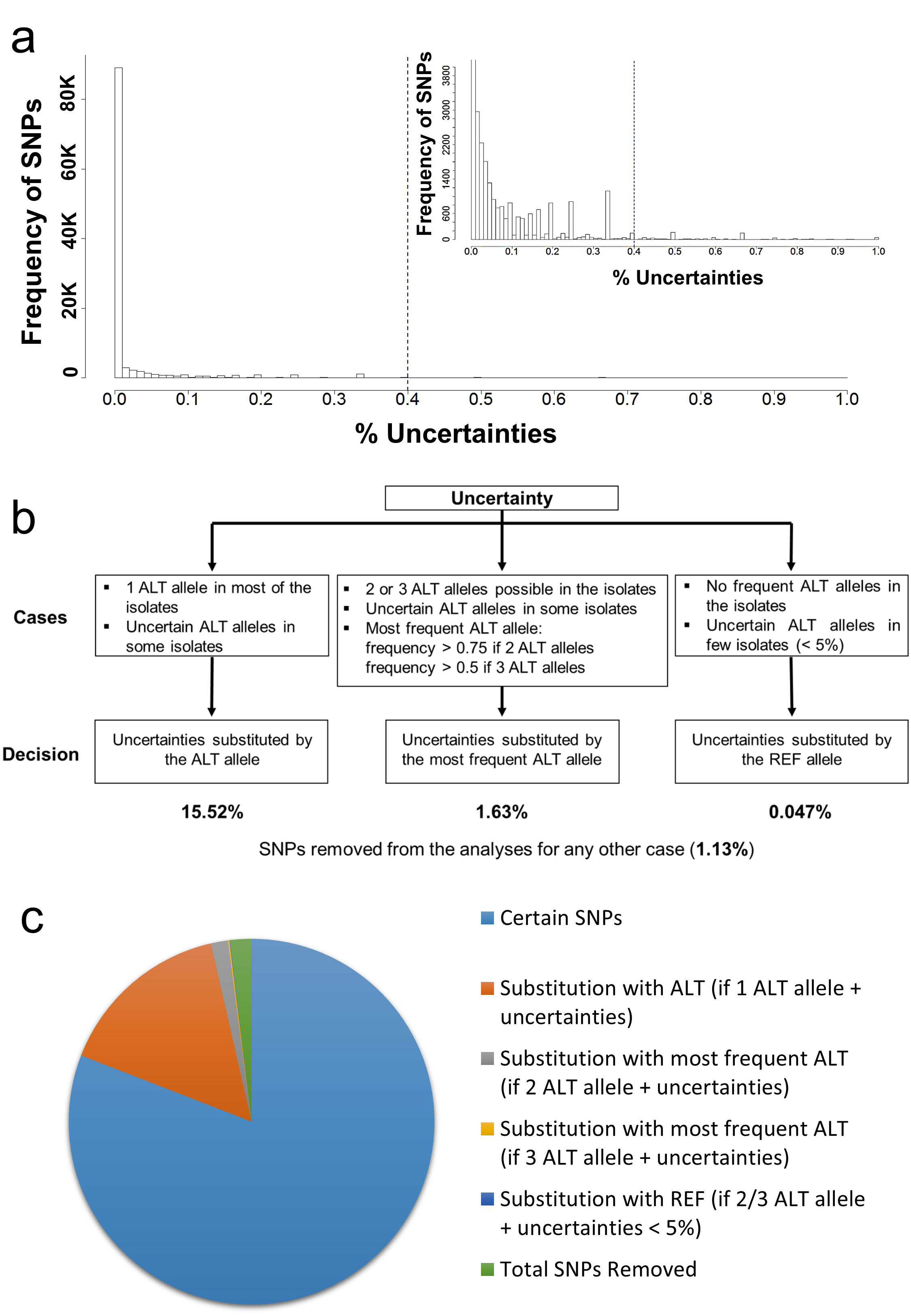

Supplement: Supplementary file 6 — Additional file 6. Criteria and statistics of SNPs with uncertain ALT alleles in the isolates. Uncertain SNPs are defined as the SNPs with more than one ALT allele in at least one of the 167 isolates. a Shows the histogram of uncertain ALT allele frequency. All the SNPs with uncertain ALT allele frequency greater than 40% (dotted blue line at 0.4) were removed from the analyses. b Represents a schematic diagram of different cases considered for SNPs with uncertainties and the decision of substitution taken. Uncertainties were substituted with the most frequent ALT value or REF value in around 17% of the SNPs at this step. c Represents the pie chart with percent of certain SNPs, substituted SNPs and removed SNPs. [file 12936_2017_2140_MOESM6_ESM.tif]

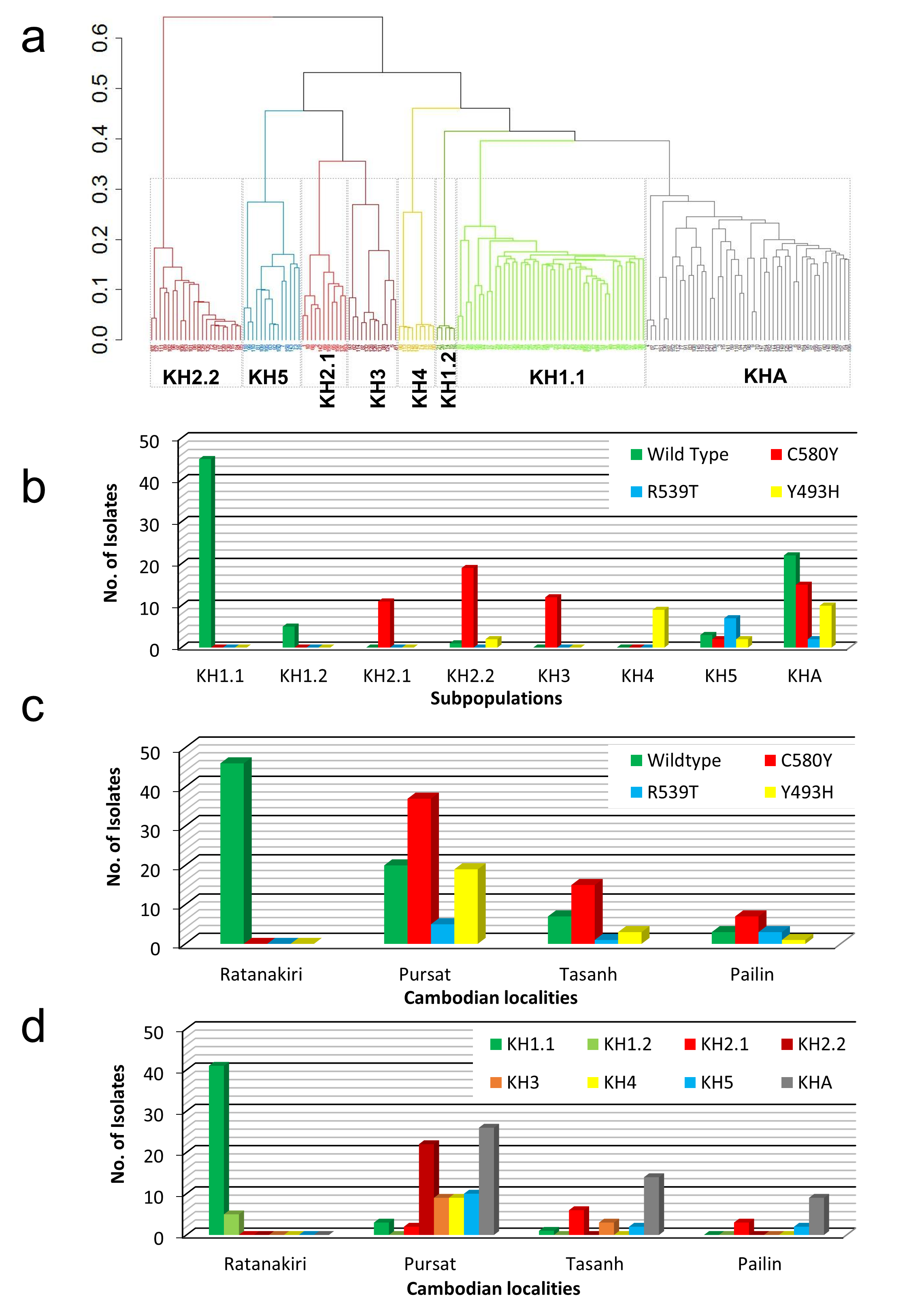

Supplement: Supplementary file 8 — Additional file 8. Classification of 167 samples into artemisinin resistant and sensitive Cambodian subpopulations. a Dendrogram representing the classification of 167 parasite isolates into 8 clusters (k = 8). The pairwise distance between two samples is calculated as the proportion of base substitution between them over the genome. Ward’s minimum variance method is used as the metric to build the dendrogram. Different clusters (subpopulations) are represented with different colors. b The barplot represents the number of isolates in each subpopulation described. “Green” represents ART-S isolates. “Red”, “Yellow” and “Blue” color represents isolates with C580Y, Y493H and R539T k13 mutations, respectively. c The barplot represents the number of isolates in each Cambodian locality (Ratanakiri, Pursat, Tasanh and Pailin) and the colors represent the type of k13 mutation present. d The barplot represents the number of isolates in each locality and the colors represent the associated subpopulation to each isolate. [file 12936_2017_2140_MOESM8_ESM.tif]

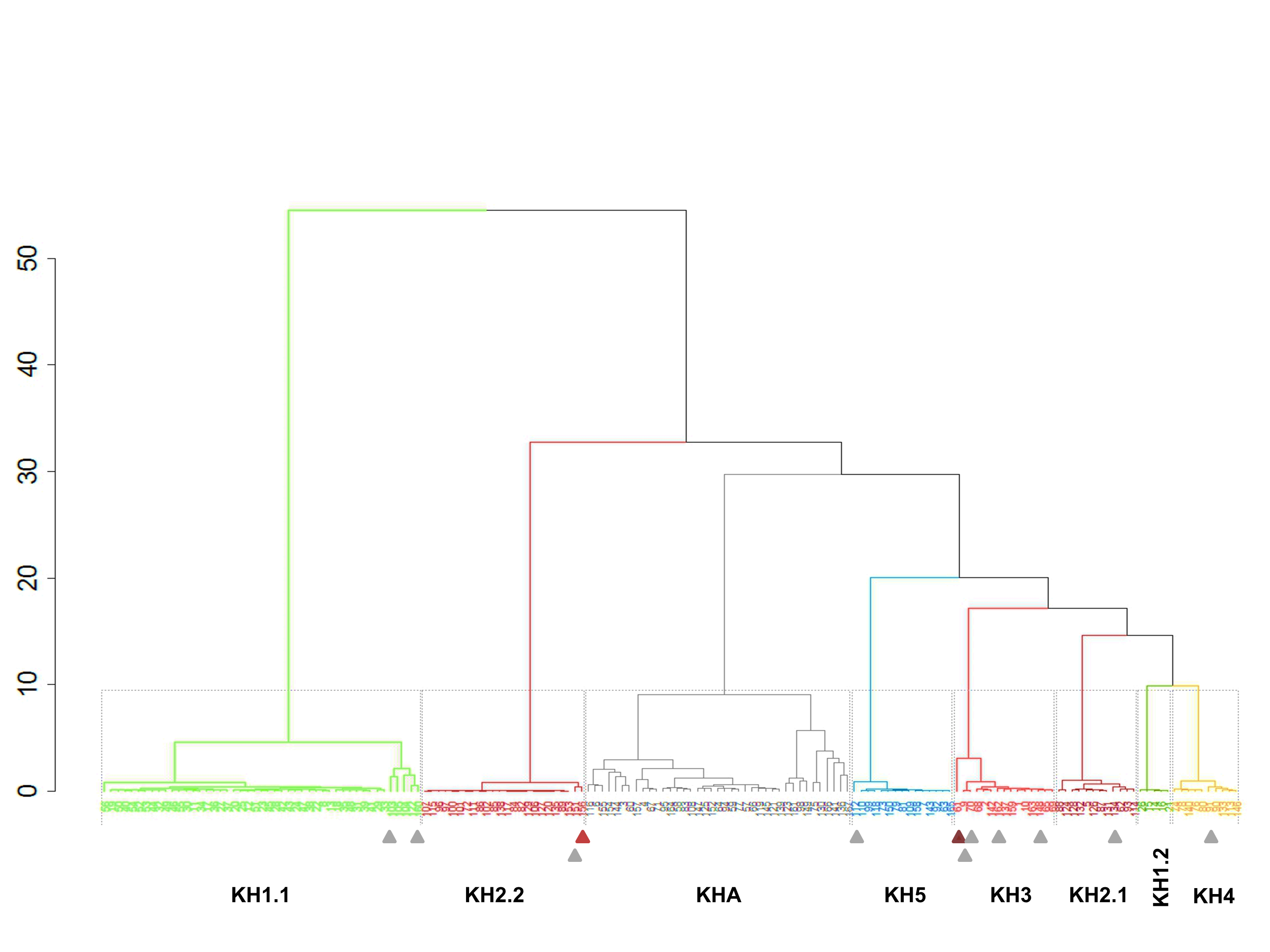

Supplement: Supplementary file 9 — Additional file 9. Hierarchical clustering of 167 isolates based on the network based stratification method [27]. Interaction network was recovered from STRINGv10 and interaction evidence from all the sources was used. Only top 10% of the interactions were included in the analysis. Similarity matrix was computed using consensus clustering, which was performed by selecting 80% mutated genes and 80% isolates 100 times randomly and iterating NMF clustering 10 times. This similarity matrix was then used to build a dendrogram for all 167 isolates using Euclidean distance matrix and ward minimum variance method in R. Colors for different clusters were assigned by comparison with the hierarchical clustering result based on 21,257 SNPs. The isolates classified in different clusters in two approaches (SNP based and Network based) are pointed with triangles. The colors of the triangles correspond to the hierarchical classification. [file 12936_2017_2140_MOESM9_ESM.tif]

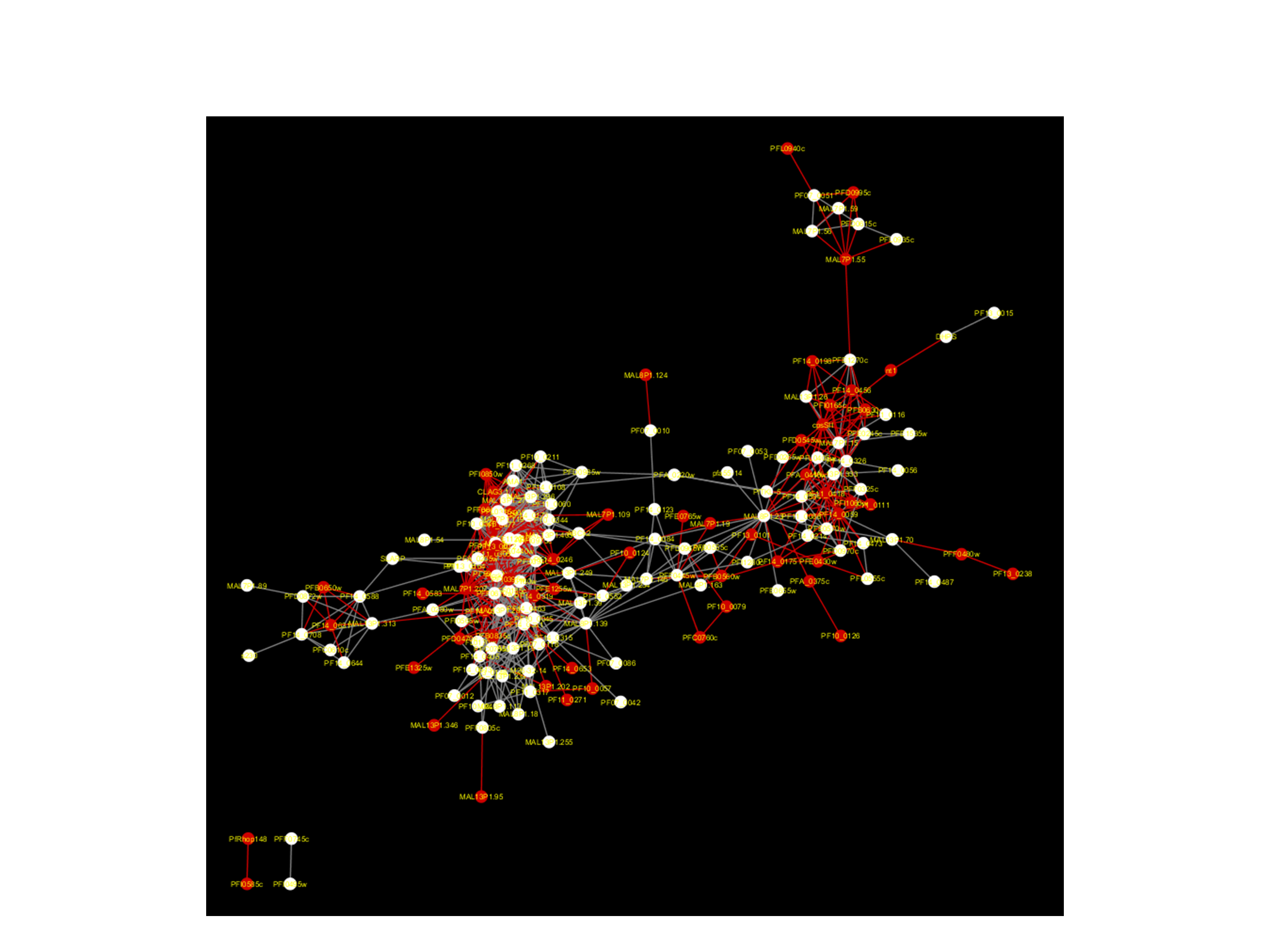

Supplement: Supplementary file 11 — Additional file 11. Network representation of overlapping gene sets associated with ART-R subpopulations (set of 265 genes) and the ART-S subpopulation KH1.2 common resistance background (set of 168 genes). The networks for the two gene sets based on co-expression data are recovered from STRING v10. The edges connecting the genes, have the co-expression evidence score greater than 0.5. The nodes and edges in “red” color represent the interaction network based on coexpression for ART-R subpopulations gene set. The nodes and edges in “white” color represent the interaction network based on coexpression for ART-S sunpopulation KH1.2 common resistance background genes set. For the ART-R subpopulation specific genes set, out of the 265 genes only 173 genes are used for overlap and for the KH1.2 common resistance background genes set, out of 168 only 113 genes are used for overlap. Other genes (nodes) are removed either because of no interactions (before/after overlap) or STRING confidence score below 0.5. The representation of overlapping coexpression network is done in Cytoscape v3.2.1 using the DyNet Analyzer plugin. [file 12936_2017_2140_MOESM11_ESM.tif]

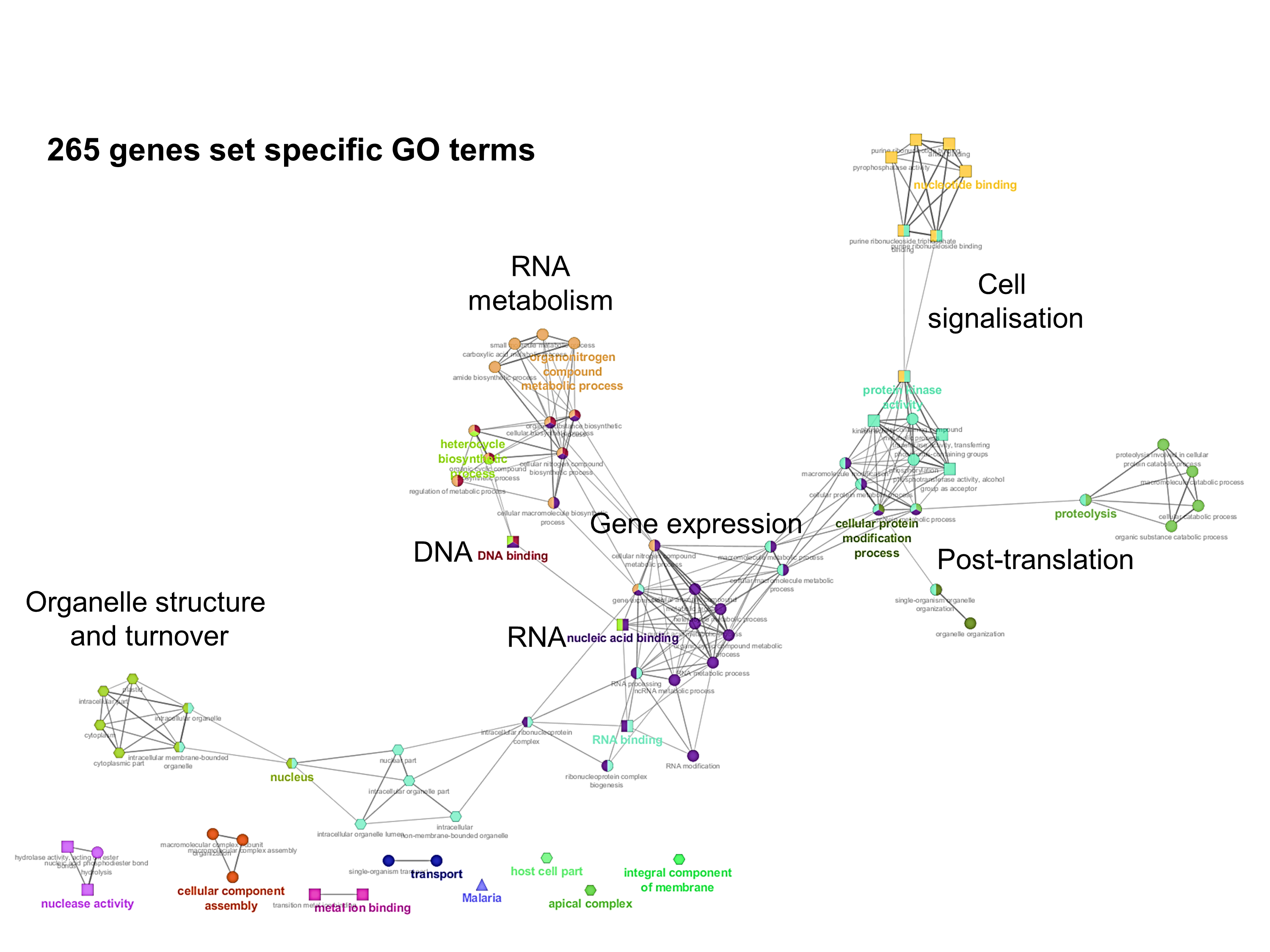

Supplement: Supplementary file 12 — Additional file 12. The functionally grouped networks of GO terms and pathways for ART-R subpopulation specific genes set (265 genes). This network represents the associations between the GO terms based on the similarity of the genes. The nodes represent the GO terms and the edges are the associations based on kappa score, which is also used for defining functional groups. Each functional group is represented with the most significant GO term in the functional group. The “Triangles” represent the metabolic pathways, “Ellipse” represents the GO terms associated to biological processes, “Hexagon” represents the GO terms associated to cellular component and the “Rectangles” represent the GO terms associated to Molecular functions. Different colors signify different GO terms functional groups. Nodes with more than one color represents the GO terms included in more than one functional group. The network of GO terms functional group is built in Cytoscape v3.2.1 using the plugin ClueGO v2.2.4. [file 12936_2017_2140_MOESM12_ESM.tif]

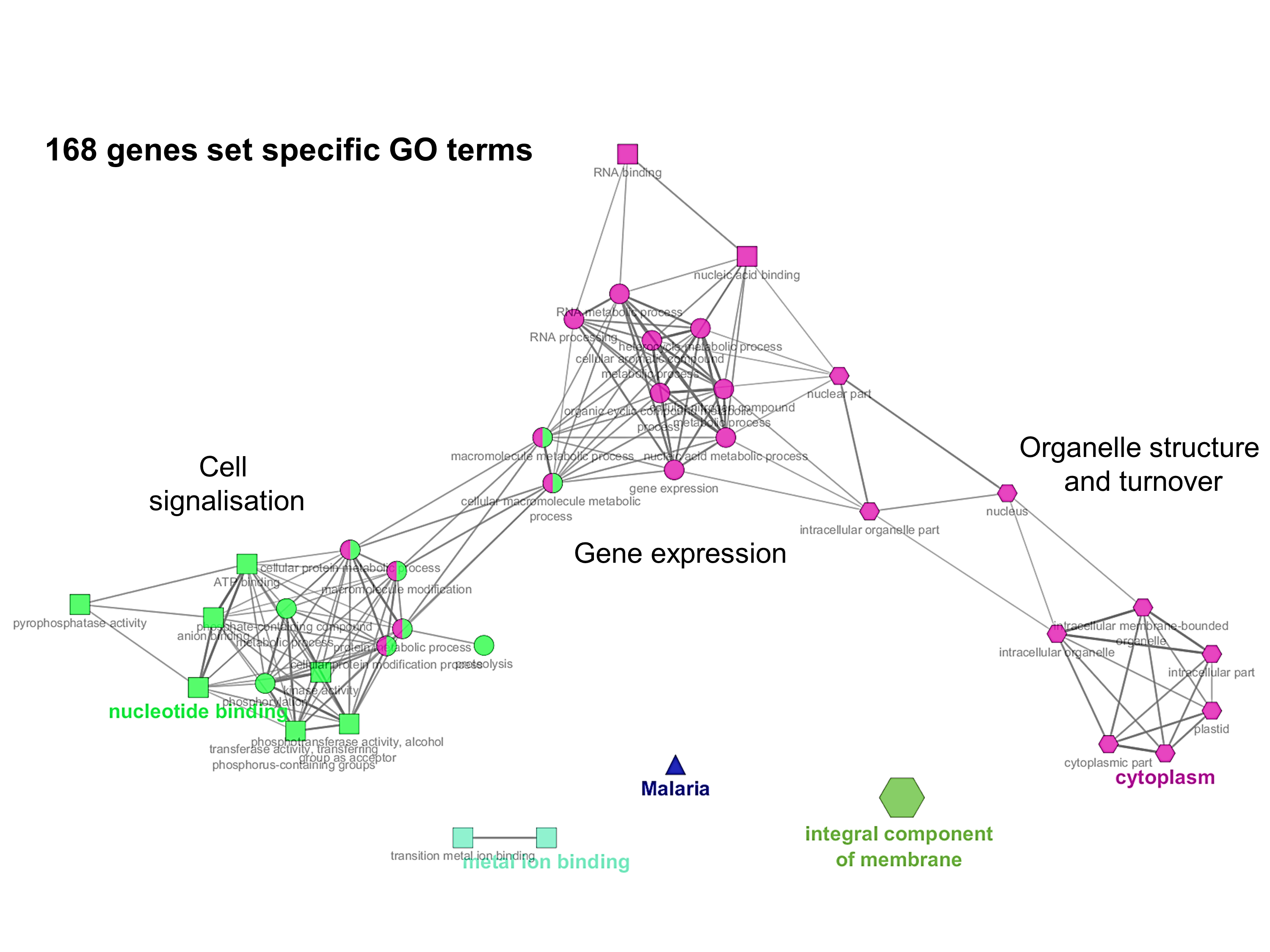

Supplement: Supplementary file 13 — Additional file 13. The functionally grouped networks of GO terms and pathways for ART-S subpopulation KH1.2 common resistance background genes set (168 genes). This network represents the associations between the GO terms based on the similarity of the genes. The nodes represent the GO terms and the edges are the associations based on kappa score, which is also used for defining functional groups. Each functional group is represented with the most significant GO term in the functional group. The “Triangles” represent the metabolic pathways, “Ellipse” represents the GO terms associated to biological processes, “Hexagon” represents the GO terms associated to cellular component and the “Rectangles” represent the GO terms associated to Molecular functions. Different colors signify different GO terms functional groups. Nodes with more than one color represents the GO terms included in more than one functional group. The network of GO terms functional group is built in Cytoscape v3.2.1 using the plugin ClueGO v2.2.4. [file 12936_2017_2140_MOESM13_ESM.tif]

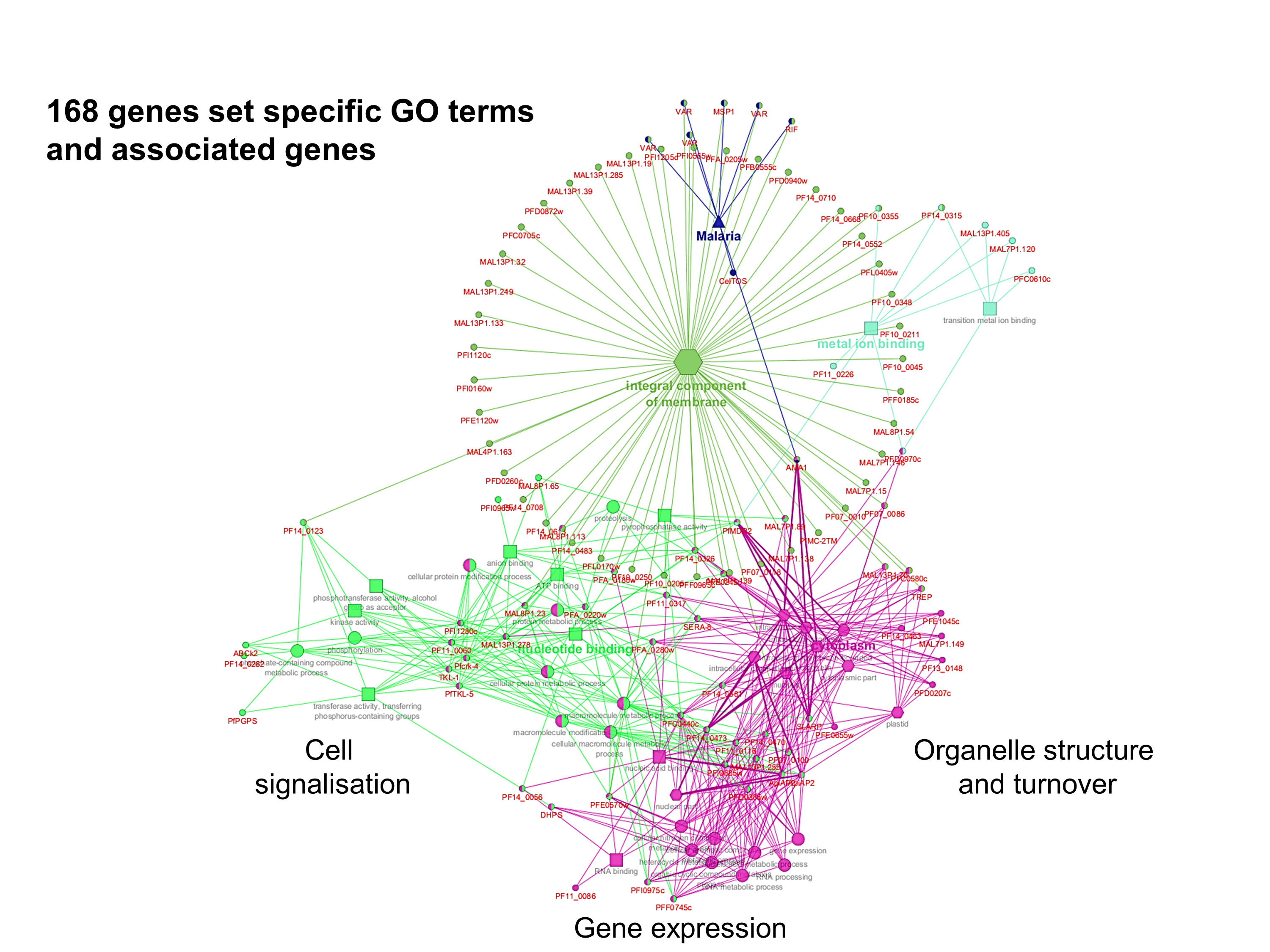

Supplement: Supplementary file 14 — Additional file 14. The functionally grouped networks of GO terms and pathways with associated genes for ART-S subpopulation KH1.2 common resistance background genes set (168 genes). This network represents the associations between the GO terms based on the similarity of the genes. The nodes represent the GO terms and the edges are the associations based on kappa score, which is also used for defining functional groups. Each functional group is represented with the most significant GO term in the functional group. The “Triangles” represent the metabolic pathways, “Ellipse” represents the GO terms associated to biological processes, “Hexagon” represents the GO terms associated to cellular component and the “Rectangles” represent the GO terms associated to Molecular functions. Different colors signify different GO terms functional groups. Nodes with more than one color represents the GO terms included in more than one functional group. The network of GO terms functional group with associated genes is built in Cytoscape v3.2.1 using the plugins ClueGO v 2.2.4 and CluePedia v 1.2.4. [file 12936_2017_2140_MOESM14_ESM.tif]

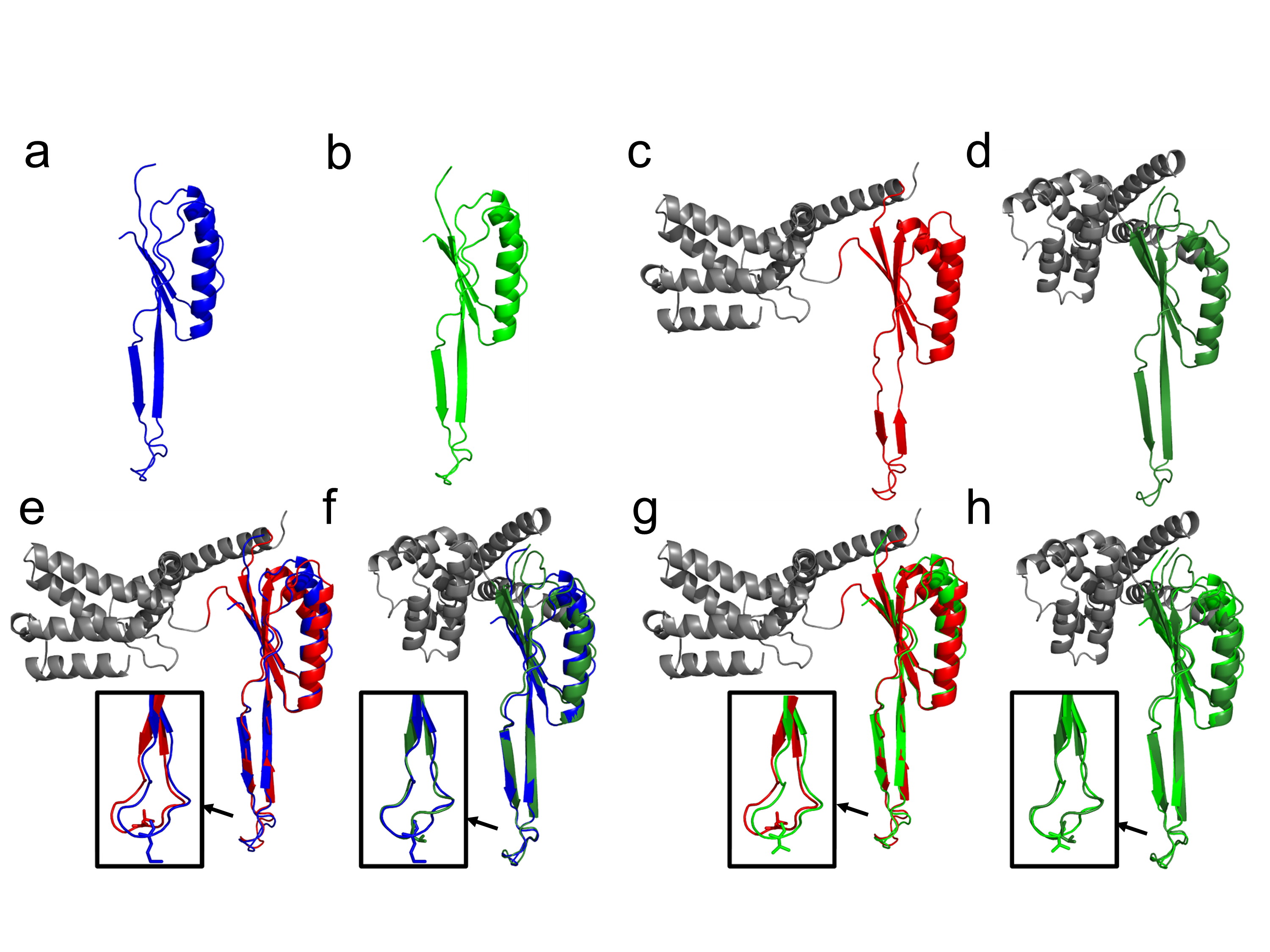

Supplement: Supplementary file 15 — Additional file 15. Structural comparison of 30S ribosomal protein S10 protein models from Neisseria gonorrhoeae, Neisseria meningitidis and Plasmodium falciparum, which can mediate tetracycline resistance. a Model of the N. gonorrhoeae 30S ribosomal S10 protein (NCBI GI#501495768), colored in blue. b Model of the N. meningitidis 30S ribosomal S10 protein (NCBI GI#488148952), in green. c Model of the P. falciparum PF3D7_1460900.1 protein, with the central domain colored in red, with two N-terminal (residues 1–68) and C-terminal (residues 180–274) domains colored grey. d Model of the P. falciparum PF3D7_1460900.2 protein, with the central domain colored in forest green, with two N-terminal (residues 1–68) and C-terminal (residues 176-268) domains colored grey. e Shows the superposition of a (blue) and c (red), with a TM-score of 0.8549. f Shows the superposition of a (blue) and d (forest green), with a TM-score of 0.8736. g Shows the superposition of b (green) and c (red), with a TM-score of 0.8610. h Shows the superposition of b (green) and d (forest green), with a TM-score of 0.8786. Proteins models were constructed using the RaptorX server [40]. TM-score and protein superposition determined using TM-align [43]. TM-scores above 0.5 indicate the proteins have the same fold. All images were rendered in PyMOL. A zoom in the loop containing the putative tetracycline resistance mutation is inserted in e–h panels. [file 12936_2017_2140_MOESM15_ESM.tif]

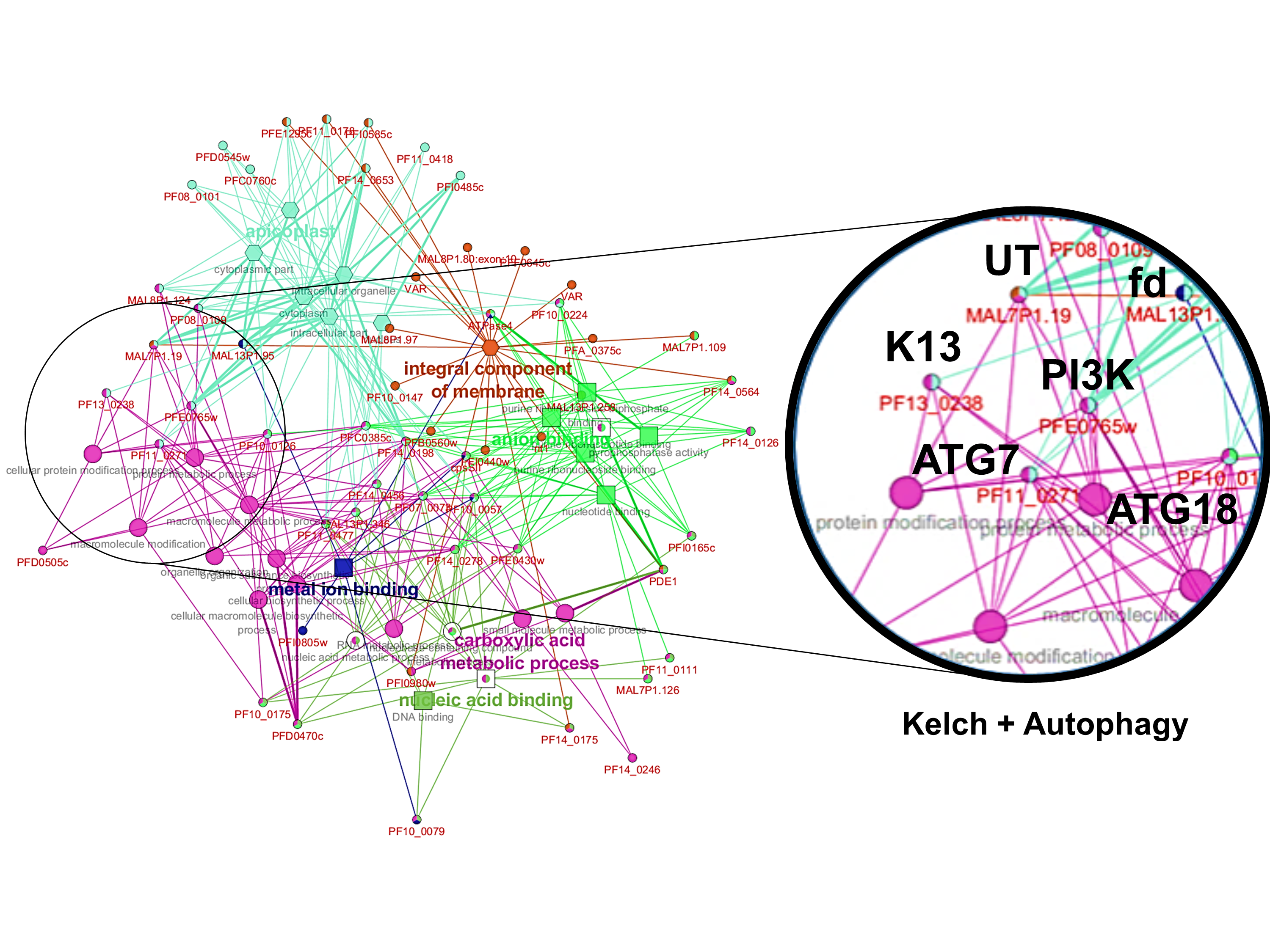

Supplement: Supplementary file 16 — Additional file 16. The functionally grouped networks of GO terms and pathways with associated genes for ART-R subpopulations specific genes set (97 genes). This network represents the associations between the GO terms based on the similarity of the genes. The nodes represent the GO terms and the edges are the associations based on kappa score, which is also used for defining functional groups. Each functional group is represented with the most significant GO term in the functional group. The “Triangles” represent the metabolic pathways, “Ellipse” represents the GO terms associated to biological processes, “Hexagon” represents the GO terms associated to cellular component and the “Rectangles” represent the GO terms associated to Molecular functions. Different colors signify different GO terms functional groups. Nodes with more than one color represents the GO terms included in more than one functional group. Different parts of the network are marked with general annotation terms. The region with genes closely associated to k13 gene (PF13_0238) based on GO terms are marked with a circle. Genes are also associated to autophagy (PF11_0271 and PF10_0126). The network of GO terms functional group with associated genes is built in Cytoscape v3.2.1 using the plugins ClueGO v 2.2.4 and CluePedia v 1.2.4. [file 12936_2017_2140_MOESM16_ESM.tif]

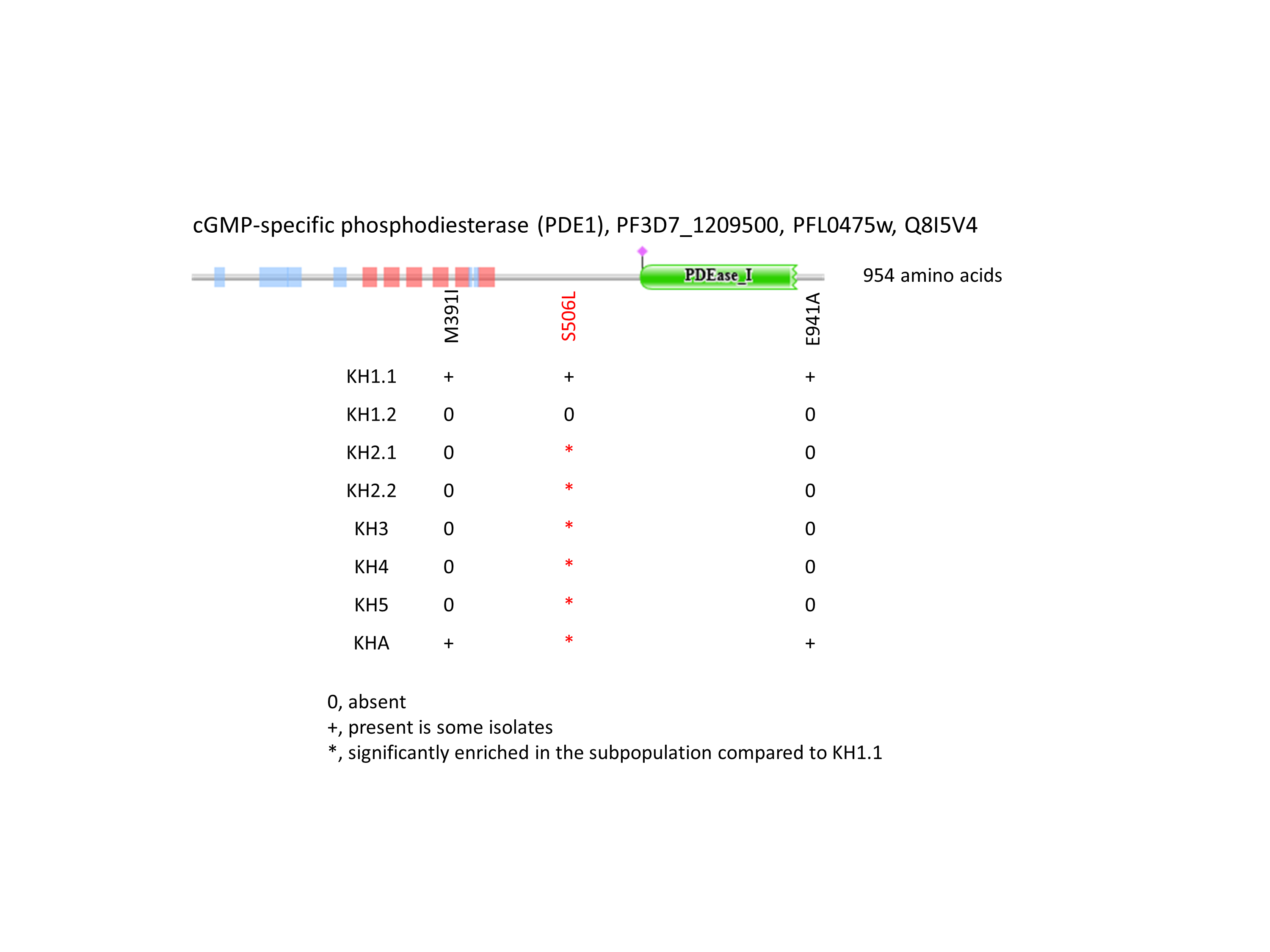

Supplement: Supplementary file 17 — Additional file 17. Distribution of significant mutation in PDE1 protein of P. falciparum in described Cambodian subpopulations. Protein description was generated using Pfam database web server. Amino acid substitution and position in the protein are given for the three mutations. [file 12936_2017_2140_MOESM17_ESM.tif]

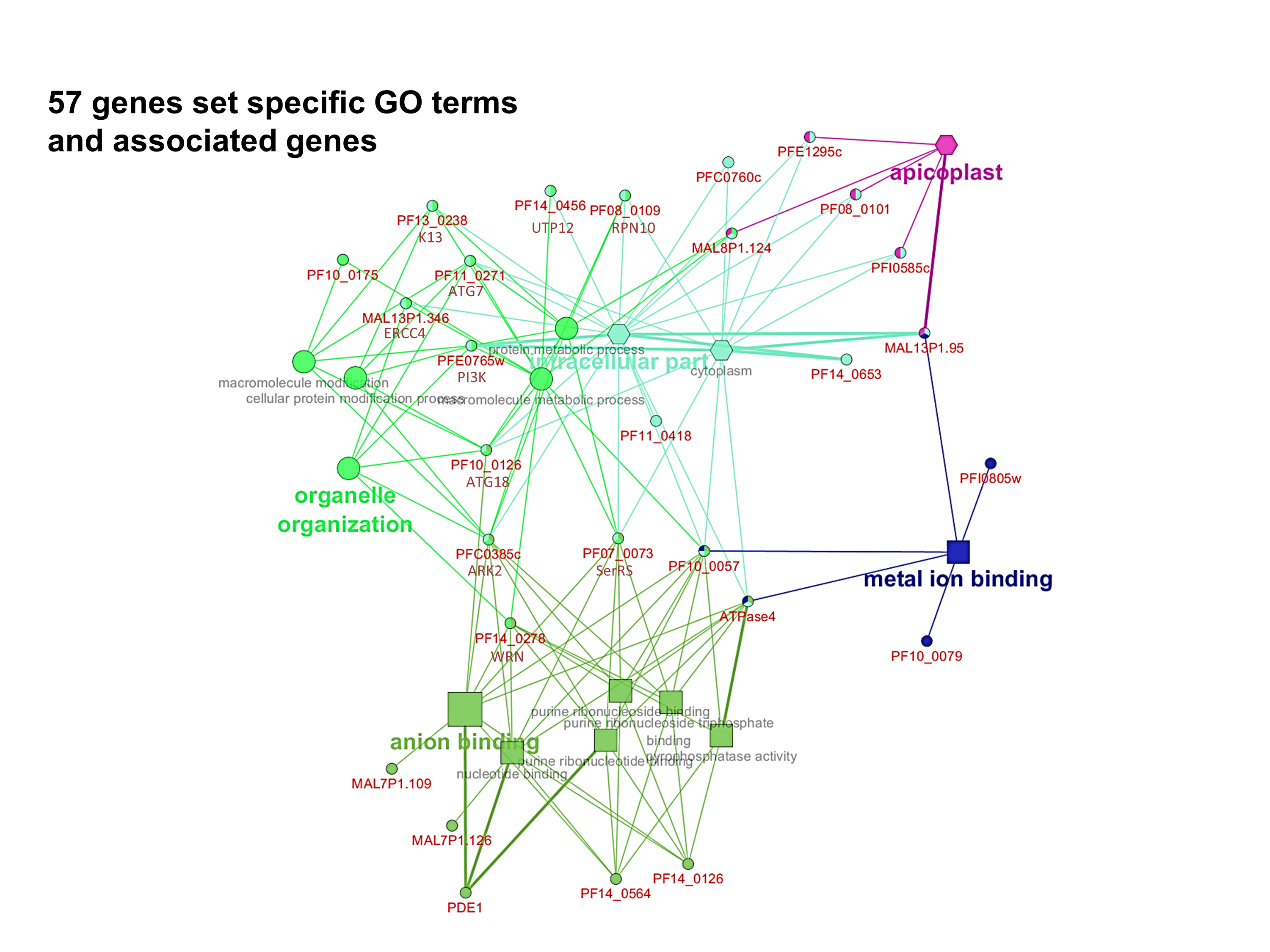

Supplement: Supplementary file 18 — Additional file 18. The functionally grouped networks of GO terms and pathways with associated genes for artemisinin resistance background genes set (57 genes), obtained by overlapping KHA specific genes (467 genes) and ART-R subpopulations specific genes (97 genes). This network represents the associations between the GO terms based on the similarity of the genes. The nodes represent the GO terms and the edges are the associations based on kappa score, which is also used for defining functional groups. Each functional group is represented with the most significant GO term in the functional group. The “Triangles” represent the metabolic pathways, “Ellipse” represents the GO terms associated to biological processes, “Hexagon” represents the GO terms associated to cellular component and the “Rectangles” represent the GO terms associated to Molecular functions. Different colors signify different GO terms functional groups. Nodes with more than one color represents the GO terms included in more than one functional group. The network of GO terms functional group is built in Cytoscape v3.2.1 using the plugin ClueGO v2.2.4. [file 12936_2017_2140_MOESM18_ESM.tif]

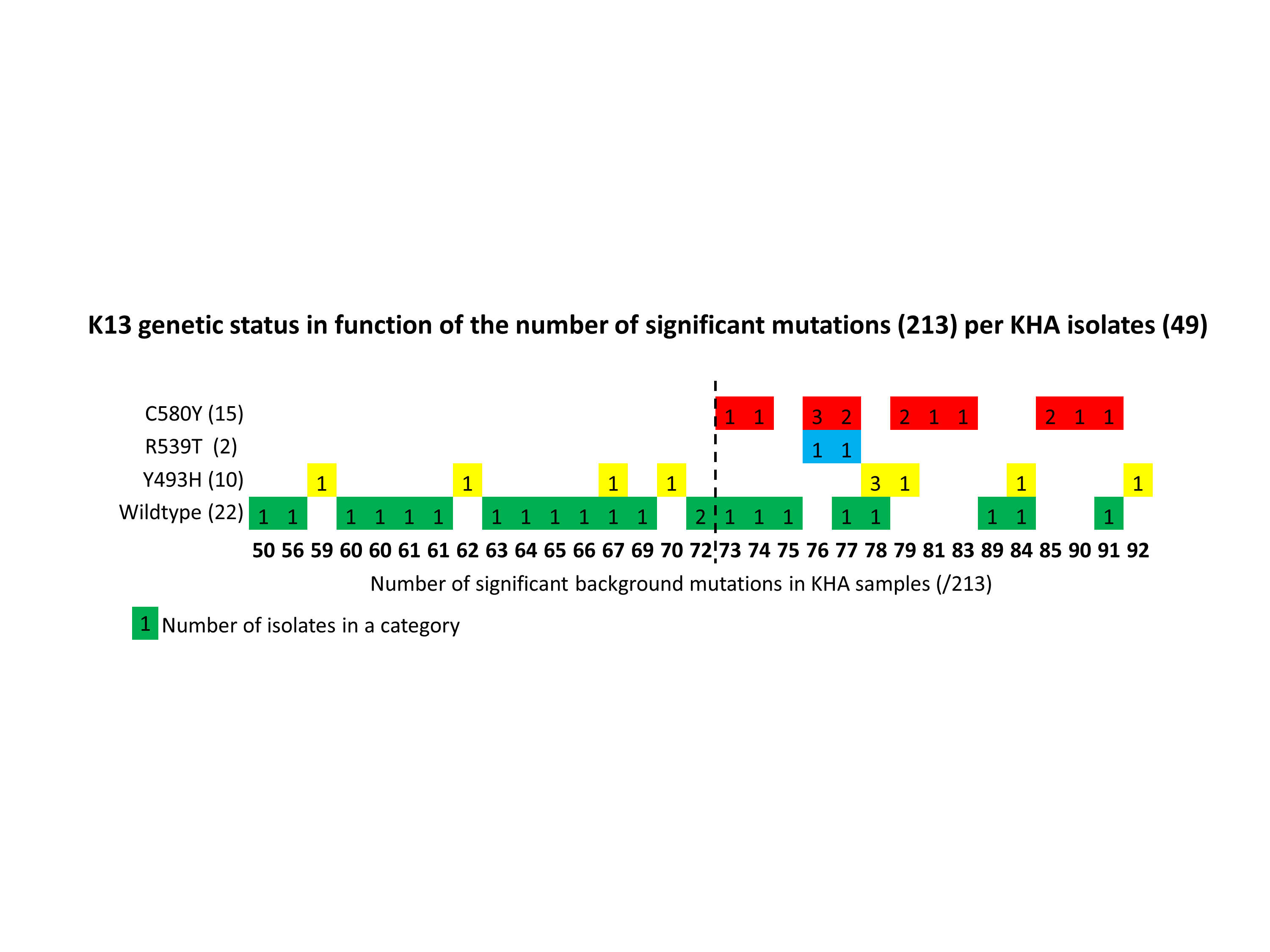

Supplement: Supplementary file 19 — Additional file 19. Relationship between k13 alleles and the number of significant mutations in the 57 genes occurring by diffusion in the KHA isolates according to ESD model. A set of 213 mutations were found in common between KHA significant mutations and in at least one ATR-R subpopulation. Horizontal axis corresponds to the number of mutations among the 213 mutations set found in one isolate. Vertical axis refers to the k13 alleles. Matrix gives the number of isolates with corresponding genetic features. Color code refers to k13 alleles: green, wildtype; red, C580Y; blue, R539T; yellow, Y493H. [file 12936_2017_2140_MOESM19_ESM.tif]

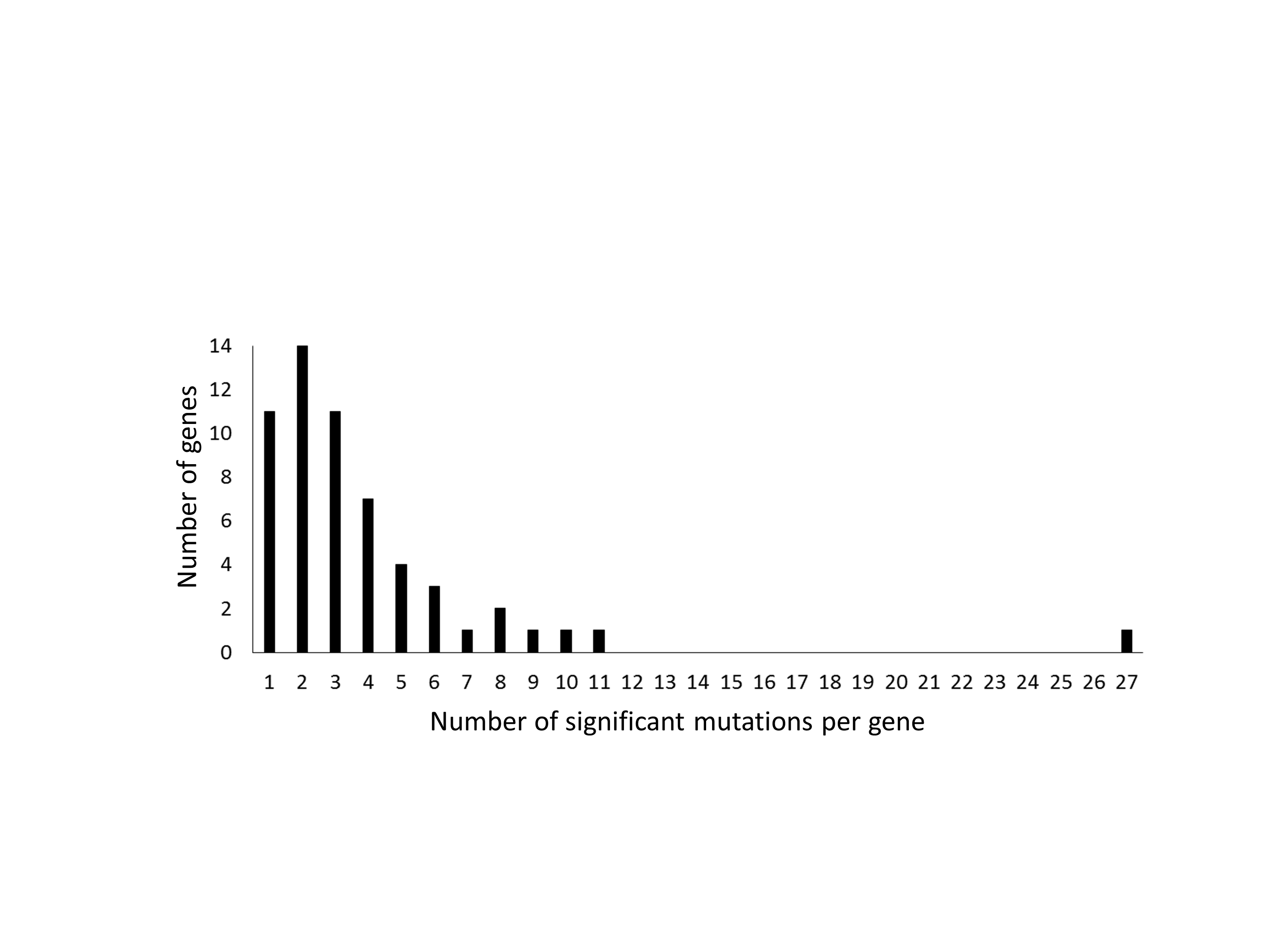

Supplement: Supplementary file 20 — Additional file 20. Distribution of the 213 significant mutations supporting the 57 background genes. These mutations are found in common between KHA significant mutations and those found in at least one ATR-R subpopulation. They are suspected to be present in the KHA subpopulation after diffusion, by crossing with parasites from the ART-R subpopulations. [file 12936_2017_2140_MOESM20_ESM.tif]
